# Diurnal Glycemic Patterns during an 8-Week Open-Label Proof-of-Concept Trial of Empagliflozin in Type 1 Diabetes

**DOI:** 10.1371/journal.pone.0141085

**Published:** 2015-11-06

**Authors:** Bruce A. Perkins, David Z. I. Cherney, Nima Soleymanlou, Justin A. Lee, Helen Partridge, Holly Tschirhart, Bernard Zinman, Roger Mazze, Nora Fagan, Stefan Kaspers, Hans-Juergen Woerle, Uli C. Broedl, Odd Erik Johansen

**Affiliations:** 1 Department of Medicine, Division of Endocrinology and Metabolism, University of Toronto, Toronto, ON, Canada; 2 Department of Medicine, Division of Nephrology, Toronto General Hospital, University Health Network, University of Toronto, Toronto, ON, Canada; 3 Boehringer Ingelheim Canada Ltd./Ltée, Burlington, ON, Canada; 4 Lunenfeld-Tanenbaum Research Institute, Mount Sinai Hospital, Toronto, ON, Canada; 5 WHO Collaborating Center, Minneapolis, Minnesota, United States of America; 6 Boehringer Ingelheim Pharmaceuticals, Inc., Ridgefield, Connecticut, United States of America; 7 Boehringer Ingelheim Pharma GmbH & Co.KG, Ingelheim, Germany; 8 Boehringer Ingelheim Norway K.S, Asker, Norway; Vanderbilt University, UNITED STATES

## Abstract

**Background:**

We recently reported improved glycemic control with reduced insulin dose in subjects with type 1 diabetes treated with the sodium glucose co-transporter-2 inhibitor empagliflozin. To further characterize the effects, we analyzed diurnal glycemic patterns by continuous glucose monitoring (CGM).

**Methods:**

In an 8-week single-arm open-label pilot study of empagliflozin, we compared ambulatory glucose profiles produced from CGM data during 2-week intervals in a placebo run-in baseline period, end-of-treatment, and post-treatment. Change in glycemic exposure was evaluated by area under the median curve according to time of day (AUC_TOTAL_ 12:00am-11:55pm; AUC_DAY_ 7:05am-10:55pm, AUC_NIGHT_ 11:00pm-7:00am), as well as glycemic variability, glycemic stability and time-in-target (≥70 to ≤140mg/dL).

**Results:**

The 40 patients (26 on insulin pump) were aged 24±5 years and BMI 24.5±3.2 kg/m^2^. Consistent with the observed HbA1c decrease (8.0±0.9% to 7.6±0.9%, p<0.0001), normalized AUC_TOTAL_ CGM decreased from 153.7±25.4 to 149.0±30.2mg/dL∙h at end-of-treatment (p = 0.31), and significantly increased post-treatment (164.1±29.5mg/dL∙h, p = 0.02). The numerical decrease in normalized AUC_NIGHT_ (152.0±36.6 to 141.9±34.4mg/dL∙h, p = 0.13) exceeded AUC_DAY_ (154.5±24.5 to 152.6±30.4mg/dL∙h, p = 0.65). Trends toward lower glycemic variability (83.1±18.9 to 75.6±28.6mg/dL, p = 0.06) and little change in glycemic stability (10.8±3.6 to 10.3±4.5mg/dL/h, p = 0.51) were observed. When empagliflozin was discontinued, these worsened relative to baseline (89.3±19.3mg/dL, p = 0.04 and 11.8±3.7mg/dL/hr, p = 0.08). Time-in-target numerically increased (40.2±11.9 to 43.1±13.5%, p = 0.69) at end-of-treatment but reversed post-treatment. Findings were similar on stratification of pump and MDI subjects.

**Conclusions:**

We observed that empagliflozin was associated with patterns of improved nighttime glycemia more prominent than daytime.

**Trial Registration:**

Clinicaltrials.gov NCT01392560

## Introduction

Despite the advent of new therapeutic strategies and technologies to improve type 1 diabetes mellitus (T1DM) management, target glycemic control is not systematically achieved and further optimization of therapy is hindered by hypoglycemia and weight gain.[1–5] Akin to studies in type 2 diabetes,[6–8] sodium glucose cotransporter-2 (SGLT2) inhibitors may be effective as adjunctive-to-insulin therapy in T1DM to favourably improve glycemic control, hypoglycemia risk, and the weight gain associated with over-insulinization.[9–15]

A potential clinical question linked to SGLT2 inhibitor therapy as adjunctive-to-insulin in T1DM is the degree of possible insulin dose adjustment that may be required upon initiation of treatment to minimize a potential hypoglycemia risk. Though the decision to adjust insulin doses at initiation is clearly influenced by specific patient characteristics such as level of glycemic control and current frequency of hypoglycemia, inherent antihyperglycemic features of SGLT2 therapy in patients with T1DM requires further analysis. From a clinical perspective, it remains to be determined whether the antihyperglycemic effect is most prominent during the day when post-prandial glycemic variability occurs, or whether the effect is prominent during nighttime hours when subjects are primarily fasted. This knowledge could inform the design of insulin dose adjustment in randomized control trials and the eventual application of adjunctive SGLT2 inhibitor therapy in T1DM clinical practice.

In the pivotal 8-week single arm, open-label adjunctive-to-insulin renal mechanistic pilot trial of empagliflozin in 40 subjects with T1DM, improvements in HbA1C, hypoglycemic risk, and weight loss were observed.[10, 16, 17] To further characterize the glycemic effects of empagliflozin, we explored diurnal glycemic patterns from interstitial fluid enzyme-based CGM using analysis by ambulatory glucose profiles (AGP).[18] Additionally, we evaluated these diurnal patterns stratified according to insulin pump and multiple daily injection (MDI) subgroups.

## Methods

The methods of the open-label 8-week Adjunctive-To-Insulin and Renal MechAnistic pilot trial (ATIRMA trial) have previously been published,[10, 16, 17, 19] and the study protocol is included in supplemental materials to this manuscript (**S1 Protocol**). In brief, we recruited normotensive, normoalbuminuric adult patients with T1DM and HbA1C 6.5–11.0% (48–97 mmol/mol). Glycemic measures in this trial were exploratory, while renal outcomes represented the primary analysis.[10, 16] Subjects underwent a 2-week placebo run-in period, 8-week treatment period with open-label empagliflozin 25 mg oral once daily, and 2-week post-treatment follow-up period. Participants documented daily capillary glucose, carbohydrate intake, and insulin doses. Given anticipated increases in daily urinary glucose excretion (by 80–90 g, which represents approximately one-third of typical daily carbohydrate intake), prandial insulin was reduced by 30%.[20] As an additional safety measure, basal insulin was reduced by 30%.[20] Subsequent insulin dose adjustments were performed under investigator guidance based on capillary glucose measurements. Recruitment began in July 2011, and the study took place from August 2011 to August 2012. The protocol and consent procedures were conducted in accordance with the World Medical Association’s Helsinki Declaration and were approved by the local Research Ethics Board at the University Health Network (Toronto, ON, Canada) prior to participant recruitment; all subjects gave informed written consent prior to start of study procedures. The study was registered in the Clinicaltrials.gov registry with the unique identifier NCT01392560, and the authors confirm that all ongoing and related trials for this drug/intervention are registered

### Ambulatory Glucose Profile Data Handling

Subjects used unblinded continuous glucose monitoring (Sof-Sensor electrochemical sensors, Sen-serter insertion device, MiniLink radio frequency transmitter, Guardian REAL-Time Continuous Glucose Monitoring System [Medtronic], and Contour Link Blood Glucose Meter [Bayer]) throughout the study. CGM results were analyzed and averaged over 2-week AGP assessment periods to provide modal day estimates of pre-specified conventional parameters of glycemic exposure, glycemic variability, and glycemic stability during the 2-week placebo run-in period (baseline), weeks 7 and 8 (end-of-treatment) and the two week period following discontinuation of empagliflozin (post-treatment).[18, 21, 22] Glycemic Exposure was represented by the pre-specified area under the median curve of sensor glucose readings (AUC). AUC_TOTAL_ was calculated as the sum of 24 hourly median AUC and reported normalized in units of mg/dL*hr and stratified according to daytime (AUC_DAY_, 7:05am-10:55pm) and nighttime (AUC_NIGHT_, 11:00pm-7:00am) periods.[18, 21, 22] Glycemic variability was represented as the pre-specified mean interquartile range (IQR, mg/dL) for each two week study period, in which lower values represent less variability.[18, 21, 22] Glycemic stability was determined through calculation of the pre-specified mean absolute hourly rate of change in the median curve (mg/dl/hr). As such, it is a reflection of the fluctuations in glucose levels throughout the day with lower values indicating better glycemic stability. Time in target, hypoglycemic, and hyperglycemic range were determined by tabulation of the percentage of time spent in target (≥70 to ≤140mg/dL), hypoglycemia (<70mg/dL), and hyperglycemia (>180mg/dL) ranges.

### Statistical Analysis

All analyses were performed using SAS System Version 9.3 (SAS Institute, Cary, NC). Data are presented as means ± standard deviation throughout the manuscript. Analysis of AGP parameters included evaluable data from baseline and end-of-treatment periods for 39 subjects. The post-treatment period included 38 evaluable subjects due to missing data from a subject in the MDI group. Comparisons of AGP parameters were performed using paired student’s t-tests between baseline and each of the subsequent AGP assessment periods. Statistical significance was defined by p-value <0.05. As part of an ad-hoc sensitivity analysis, in addition to the three pre-specified measures of glycemic exposure, glycemic variability, and glycemic stability described above, other CGM metrics were calculated as alternatives to the AGP. Mean sensor glucose (measured in mg/dL) was calculated as an alternative measurement of glycemic exposure. The standard deviation of all glucose instances (SD, mg/dL) and the coefficient of variation (%CV, %) were calculated as alternative measurements of glycemic variability. As an alternative measurement of glycemic stability, the mean amplitude of glycemic excursions (MAGE, mg/dL) was calculated. MAGE, originally proposed by John Service and colleagues at Mayo Clinic, was not designed for CGM data, and a modified algorithm was used for the determination of MAGE, as outlined in **S1 File**. Due to limitations in its calculation using CGM, 23 participants were included in the MAGE analysis. We note that IQR takes into account the 14-day CGM period, while MAGE uses only one representative day selected a priori; additionally, while IQR is not affected by standard deviation, MAGE is dependent on SD for its calculations.

## Results

**Fig 1:**
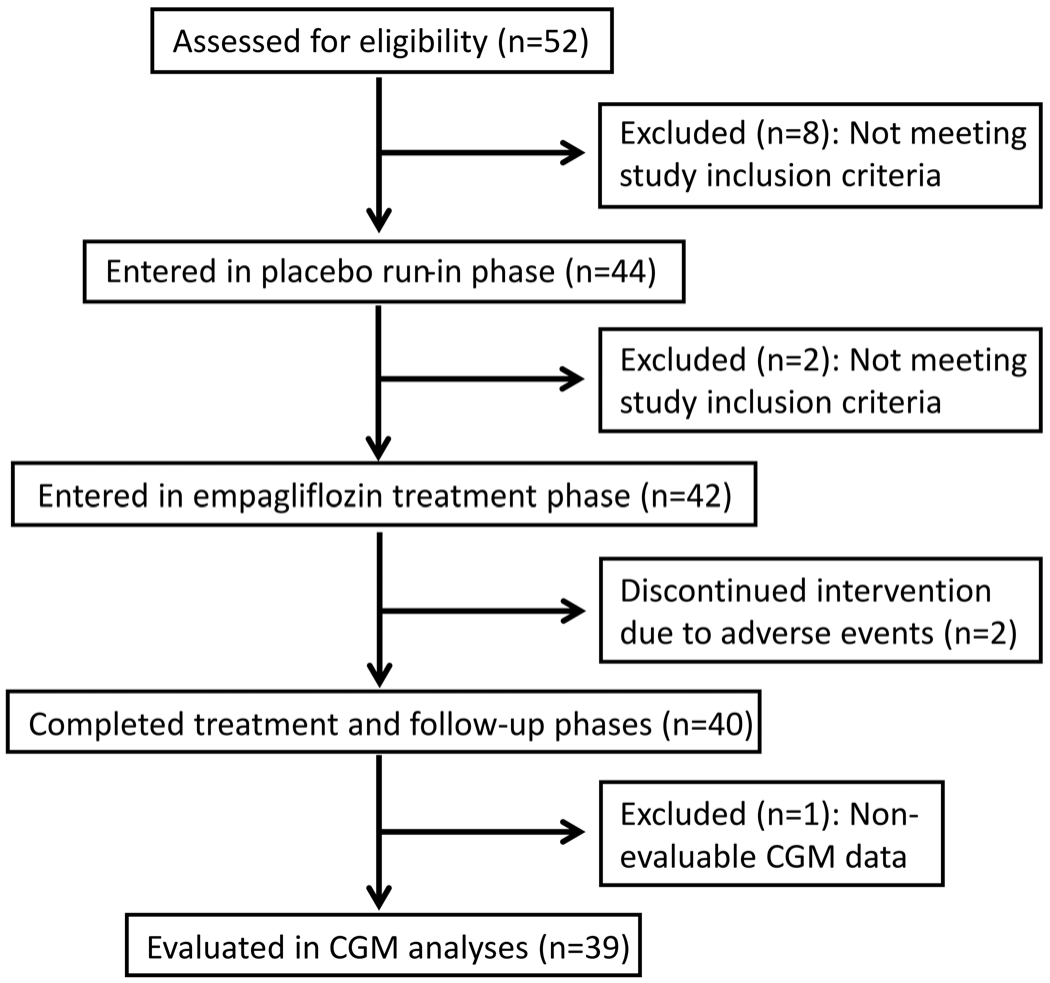
Flow chart for study participants.

The study flow chart is shown in Fig 1. The clinical characteristics of the 40 study subjects are presented in the first section of Table 1, and are also stratified according to insulin pump (n = 26; 65%) and MDI (n = 14; 35%) subgroups. The second section of Table 1 summarizes the baseline, end of treatment and follow-up glycemic measures. In comparing baseline to end-of-treatment and baseline to post-treatment effects, as previously reported[10] we found that HbA1c improved from 8.03±0.91 to 7.62±0.15% over the 8-week baseline to end-of-treatment periods (p<0.0001) and remained lower in the 2-week post-treatment interval at 7.74±0.16% (p = 0.007). A similar pattern was seen among members of the pump subgroup, while members of the smaller MDI subgroup showed a reduction at end-of-treatment that was similar in magnitude but not statistically significant, and there was a subsequent non-significant rise in HbA1c post-treatment.

**Table 1:** Characteristics of the 40 Subjects with Type 1 Diabetes.

	Total Cohort (N = 40)	Insulin Pump Subgroup (n = 26)	MDI Subgroup (n = 14)
*Clinical Characteristics*			
Male Sex, N (%)	20 (50)	13 (50)	7 (50)
Age	24.3 ± 5.1	24.8 ± 5.3	23.2 ± 4.6
Diabetes Duration			
>1 to 5 years	4	2	2
>5 years	36	24	12
Total Daily Insulin (units)	54.7 ± 20.4	53.9 ± 19.8	56.3 ± 22.3
BMI (kg/m^2^)	24.5 ± 3.2	24.7 ± 3.6	24.2 ± 2.3
Glycemic Measures*			
Baseline Fasting Glucose (mg/dL)	180.18 ± 87.66	164.88 ± 66.24	208.62 ± 115.2
HBA1c (%)			
Baseline	8.03 ± 0.91	7.95 ± 0.83	8.16 ± 1.06
End-of-treatment	7.62 ± 0.15 †	7.52 ± 0.16 †	7.81 ± 0.31
Post-treatment	7.74 ± 0.16 †	7.57 ± 0.16 †	8.05 ± 0.34
Glycemic Exposure (mg/dL)*hr			
Baseline	153.7 ± 25.4	156.3 ± 26.1	148.3 ± 23.9
End-of-treatment	149.0 ± 30.2	151.6 ± 26.9	143.8 ± 36.7
Post-treatment	164.1 ± 29.5 †	162.9 ± 28.4	166.5 ± 32.6 †
Glycemic Variability (mg/dL)			
Baseline	83.1 ± 18.9	81.3 ± 17.8	86.7 ± 21.2
End-of-treatment	75.6 ± 28.6	75.1 ± 32.5	76.5 ± 19.7
Post-treatment	89.3 ± 19.3 †	89.2 ± 21.6 †	89.4 ± 14.7
Glycemic Stability (mg/dL/hr)			
Baseline	10.8 ± 3.6	10.1 ± 3.1	12.2 ± 4.2
End-of-treatment	10.3 ± 4.5	10.0 ± 4.6	11.0 ± 4.3
Post-treatment	11.8 ± 3.7	11.5 ± 3.8 †	12.3 ± 3.6
Percentage of Time Spent 70-140mg/dL			
Baseline	40.2 ± 11.9	39.9 ± 13.8	40.9 ± 7.1
End-of-treatment	43.1 ± 13.5	43.9 ± 14.2	41.7 ± 12.2
Post-treatment	35.0 ± 12.1	36.4 ± 12.5	32.2 ± 11.2 †
Percentage of Time Spent >180mg/dL			
Baseline	34.1 ± 14.4	34.8 ± 16.0	32.6 ± 10.8
End-of-treatment	29.1 ± 14.9	29.8 ± 15.5	27.8 ± 14.3
Post-treatment	39.8 ± 15.7	38.9 ± 15.0	41.5 ± 17.4 †
Percentage of Time Spent <70mg/dL			
Baseline	5.0 ± 4.6	3.7 ± 2.9	7.6 ± 6.2
End-of-treatment	5.2 ± 6.4	3.3 ± 2.8	9.1 ± 9.4
Post-treatment	5.0 ± 4.9	4.0 ± 3.5	6.9 ± 6.5

Unless otherwise indicated all data are presented as mean ± SD.

Comparisons are made between end-of-treatment and baseline, or post-treatment and baseline within the Total Cohort and within each of the Insulin Pump and MDI subgroups.

* For the analysis of AGP parameters, evaluable data from baseline and end of treatment periods was available for 39 subjects. The post-treatment period included 38 evaluable subjects due to missing data from a subject in the MDI group.

† Indicates p<0.05 for comparison with the corresponding baseline value. Specifically: HbA1c end-of-treatment comparison with baseline for the Total Cohort, p<0.0001; post-treatment comparison with baseline for the Total Cohort, p = 0.007; end-of-treatment comparison with baseline for the Pump Group, p<0.0001; post-treatment comparison with baseline for the Pump Group, p = 0.0007. Glycemic Exposure post-treatment comparison with baseline for the Total Cohort, p = 0.02; post-treatment comparison with baseline for the MDI Group, p = 0.01. Glycemic Variability post-treatment comparison with baseline for the Total Cohort, p = 0.04; post-treatment comparison with baseline for the Pump Group, p = 0.047. Glycemic Stability post-treatment comparison with baseline for the Pump Group, p = 0.03. Percentage of Time Spent >180mg/dL post-treatment comparison with baseline for the MDI Group, p = 0.04. Percentage of Time Spent 70-140mg/dL post-treatment comparison with baseline for the MDI Group, p = 0.003.

**Fig 2:**
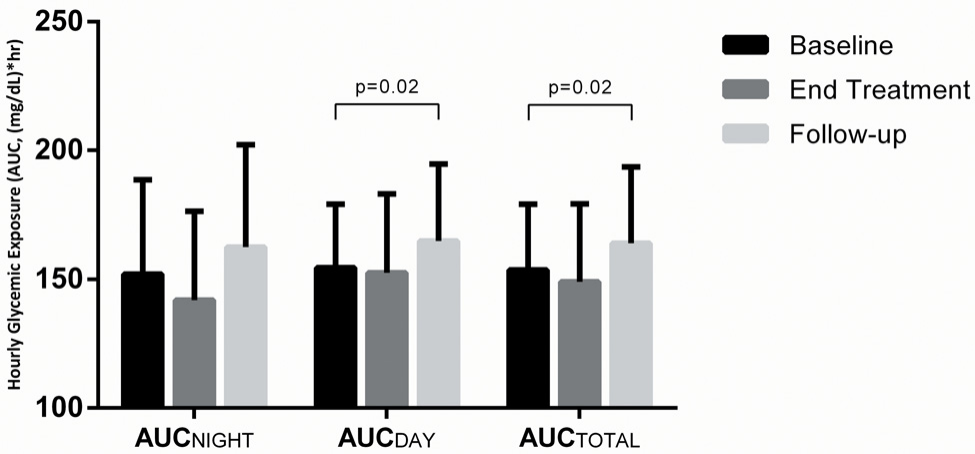
Average Hourly Glycemic Exposure According to Nighttime, Daytime, and Total Hours. Hourly glycemic exposure was evaluated by area under the median curve according to time of day: AUC_TOTAL_ 12:00am-11:55pm; AUC_DAY_ 7:05am-10:55pm, AUC_NIGHT_ 11:00pm-7:00am. P>0.05 for all comparisons except for those significant differences indicated. 25(64%) participants saw a reduction in AUC_TOTAL_ from baseline to end-of-treatment, while 14(36%) saw an increase. 28(72%) participants saw a reduction in AUC_NIGHT_ from baseline to end-of-treatment, while 11(28%) saw an increase.

Glycemic exposure, assessed over a 2-week period, demonstrated a numerical improvement from baseline to end-of-treatment. Specifically, normalized AUC_TOTAL_ numerically decreased from baseline to end-of-treatment (153.7±25.4 to 149.0±30.2 mg/dL*hr, p = 0.31), which increased significantly post-treatment to 164.1±29.5 mg/dL*hr compared to baseline (p = 0.02, Table 1). Mean sensor glucose, evaluated in sensitivity analysis as an alternative glycemic exposure measure to and highly correlated with AUC, also decreased from baseline to end-of-treatment (from 161.5±24.2 mg/dL to 155.6±28.5 mg/dL, p = 0.16), and increased post-treatment (to 171.3±28.6 mg/dL, p = 0.02). Similar patterns were observed in the insulin pump and MDI subgroups, though statistical significance for the post-treatment worsening of glycemic exposure was only observed in the MDI subgroup. To emphasize the diurnal patterns in glycemic exposure, we show the mean baseline, end-of-treatment and post-treatment AUC in Fig 2. The baseline to end-of-treatment trend in decreased glycemic exposure was associated with improvement during nighttime hours as compared to daytime hours. Secondly, the increased glycemic exposure that occurred post-treatment was observed during both nighttime and daytime hours, but statistical significance was seen specifically for the daytime post-treatment values compared to baseline (p = 0.02).

A trend toward lower glycemic variability (IQR) from baseline to end-of-treatment (83.1±18.9 to 75.6±28.6mg/dL, p = 0.06) was observed, and levels increased in the post-treatment interval relative to baseline (89.3±19.3mg/dL, p = 0.04, Table 1). This pattern was also observed in sensitivity analysis for the alternative measures of glycemic variability, though in these instances the improved variability at 8 weeks was statistically significant. Specifically, SD was 65.1±13.0mg/dL at baseline, 59.5±17.1mg/dL at end-of-treatment (p = 0.02 compared to baseline), and 68.4±13.3mg/dL post-treatment (p = 0.08 compared to baseline). Similarly, %CV was 40.5±6.5% at baseline, 38.2±7.6% at end-of-treatment (p = 0.02), and 40.3±6.75% post-treatment (p = 0.71). A similar significant pattern of change in IQR was observed among members of the pump subgroup, while members of the smaller MDI subgroup showed an increase in IQR post-treatment that was similar in magnitude but not statistically significant.

There was little evidence of a change in glycemic stability (10.8±3.6 to 10.3±4.5mg/dL/h, p = 0.51, Table 1) from the baseline interval to the end-of-treatment interval, followed by a trend to worsened stability compared to baseline in the post-treatment period (11.8±3.7mg/dL/hr, p = 0.08). In sensitivity analysis for glycemic stability, the changes in MAGE were similar. Specifically, MAGE demonstrated a non-significant decrease from 126.5±45.7mg/dL at baseline to 120.3±57.1mg/dL at end-of-treatment (p = 0.68) and rose to 130.9±51.9mg/dL post-treatment (p = 0.76).

Percentage of time spent in target range (70–140 mg/dL) showed a trend toward an increase from baseline to end-of-treatment and a trend toward a decrease post-treatment, but this post-treatment decrease in time in target was significant only for the MDI subgroup. While trend toward a decrease in the percentage of time spent in hyperglycemia (>180 mg/dL) and trend toward an increase post-treatment were observed, the post-treatment increase was only statistically significant for the MDI subgroup. Percentage of time spent in hypoglycemia did not appear to change from baseline, regardless of pump or MDI subgroups (Table 1).

Associated with these glycemic patterns were a change in the mean total daily insulin dose requirements, which we have reported previously.[10] Specifically, total daily insulin decreased from 54.7±20.4 units at baseline to 45.8±18.8 units at end-of-treatment (p<0.0001 compared to baseline), restoring to 54.2±21.1 units post-treatment. This change was mostly attributable to basal insulin, which decreased from 25.7±10.6 units at baseline to 19.5±7.9 units at end-of-treatment (p<0.0001 compared to baseline), restoring to 24.0±9.8 units post-treatment. These end-of-treatment reductions corresponded to 17% for total daily insulin and 24% for basal insulin. In contrast, mean prandial insulin delivered was 29.0±15.8 units at baseline, 27.0±14.2 units at end-of-treatment (p = 0.19 compared to baseline), and 30.2±16.1 units post-treatment. We observed (and previously reported)[10] an overall increase in carbohydrate intake from baseline to end-of-treatment, from 177±121 g/day to 229±160 g/day (p = 0.0007).

## Discussion

We observed that the improvement in HbA1c associated with 8-week adjunctive-to-insulin therapy with empagliflozin in patients with T1DM was associated with: 1) Trends toward improvement in glycemic exposure and the percentage of time spent in hyperglycemic and target ranges; 2) Significant improvement in two of the three measures of glycemic variability; 3) Trend toward improvement in glycemic exposure that was primarily explained by the improvement observed during nighttime hours despite a decrease in overnight basal insulin delivery; 4) Significant worsening in glycemic exposure and glycemic variability when empagliflozin therapy was discontinued; and 5) A similar magnitude of glycemic impact regardless of insulin pump or MDI use. Although we previously observed a significant improvement in A1c (from 8.0±0.9% at baseline to 7.6±0.9% at end-of-treatment)[10], the lack of significance in the decrease in glycemic exposure as measured by AGP may relate to the effects of a concomitant decrease in basal insulin doses and an increase in the total carbohydrate intake over the 8 weeks of study.

While CGM parameters have been explored in a 2-week T1DM trial of the SGLT2 inhibitor dapagliflozin, in which highest doses showed a trend toward improved glucose variability compared to placebo,[15] the current analysis represents a longer-term study specifically evaluating diurnal patterns in the effect of these agents. Although studies of type 1 and type 2 diabetes have shown substantial incremental benefit of different SGLT2 inhibitors on fasting glucose values,[6–8, 23–27] most studies have implied that a fundamental effect of SGLT2 inhibition is the attenuation of post-prandial glucose excursions.[28, 29] The use of the CGM-based AGP in the current study afforded a unique opportunity to explore these diurnal patterns among a cohort of patients with a range of baseline HbA1c from target levels as low as 6.5%. These results indicated that the improvement in glycemic exposure was most prominent in the interval of time from 11 pm to 7 am, when subjects were primarily asleep, fasted, and when the effect of basal rather than prandial insulin was dominant. That this improvement was accompanied by a substantial decrease in basal insulin doses over 8 weeks (from 25.7±10.6 at baseline to 19.5±7.9 units at end-of-treatment, p<0.0001; previously published[10]) implies that the improvement in nighttime glycemic exposure was a direct effect of empagliflozin therapy. To the contrary, the 2-week study of dapagliflozin found that reduction in total daily insulin was primarily a result of prandial rather than basal insulin reduction. Though we cannot explain this inconsistency between the two studies, a different insulin-adjustment strategy, the lower baseline HbA1c, and the longer duration of SGLT2 inhibitor therapy observed in the current study may account for these findings. Taken together, these two studies suggest that a reduction in total daily insulin may be needed for subjects with T1DM at the time of initiation of an SGLT2 inhibitor. The optimal level of insulin adjustment should be based inherently on patient needs at the time of adjunctive treatment. Baseline HbA1c levels and other variables such as the level of physical activity, changes in dietary adherence, and concomitant conditions are clinical considerations that may influence the magnitude of potential insulin adjustments. Assessment of glycemic status during the first few days of treatment with an SGLT2 inhibitor may be warranted by patients or care providers in order to appropriately adjust the insulin regimen based on individual requirements.

The finding of worsened glycemic exposure and glycemic variability after discontinuation of empagliflozin therapy in the current study is an important observation that supports pharmacological efficacy, particularly in light of the *a priori* insulin dose reductions. In support of the findings in the short-term randomized controlled trial,[15] worsened glycemic parameters after withdrawal–also observed with fasting capillary glucose[10] strongly supports the 8-week efficacy of empagliflozin independent of the confounding interventions in the study such as use of unblinded CGM and clinical trial participation. That differences in glycemic exposure were not systematically seen from empagliflozin therapy between the insulin pump and the MDI regimen subgroups implies that method of insulin delivery does not influence SGLT2 inhibitor efficacy.

Although this represents the first outpatient and longest SGLT2 inhibitor study in subjects with T1DM, it was limited by the restricted sample size that may not allow confident conclusions to be drawn from the stratified analysis. Furthermore, it was limited by its single arm exploratory design and by categorization of diurnal patterns based on time of day rather than on explicitly known fasted and prandial time intervals. The unbiased and continuous nature of CGM versus the episodic and biased nature of capillary glucose measurement and symptom reporting, along with their inherent differences in methodology, may explain the inconsistency between hypoglycemia reported in this analysis and the improved rates of hypoglycemia detected by capillary glucose and by symptoms during empagliflozin treatment reported previously.[10]

In summary, the previously-demonstrated impact of empagliflozin treatment on glycemic control in subjects with T1DM appears to be comparable between patients on insulin pump therapy and MDI. Furthermore, the improved glycemic control appears to result from improvement in nighttime glycemia more prominently than daytime. This novel observation may inform insulin dose adjustment in future T1DM clinical trials with SGLT2 inhibitors.

## Supporting Information

S1 Trend Checklist Transparent Reporting of Evaluations with Nonrandomized Designs statement checklist. (PDF)

S1 File Supplement describing algorithm for determination of MAGE. (DOCX)

S2 File Relevant data. (XLS)

S1 Protocol Clinical Trial Protocol. (PDF)
