# Supplementary material for: Diurnal Glycemic Patterns during an 8-Week Open-Label Proof-of-Concept Trial of Empagliflozin in Type 1 Diabetes
Source: PLoS One. 2015 Nov 6;10(11):e0141085. doi: 10.1371/journal.pone.0141085 (PMC4636141; doi:10.1371/journal.pone.0141085)
Supplement: S1 File — (DOCX) [file pone.0141085.s002.docx]

**S2. Supplement**

1. **Determination of MAGE**

The mean amplitude of glycemic excursions (MAGE) was determined using the following algorithm: Participants, in addition to using CGM, completed 8-point home blood glucose monitoring (HBGM) whereby they recorded fingerstick glucose measurements at 8 specified points throughout one day during each of the baseline, end-of-treatment, and post-treatment periods. MAGE, originally proposed by John Service and colleagues at Mayo Clinic, was not designed for CGM data. It uses the arbitrary cut-off point of one standard deviation from the mean following a meal to determine the amount of glucose variability and excess glucose exposure. Although in our previous studies we found no clinical or research purpose, we carried out further extensive research in the use of MAGE in other studies. Unable to obtain the software from investigators who employed versions of MAGE in their research, we have employed our own algorithm, which has been verified.

The rules for the MAGE determination are as follows:

1. Make all calculations based on a single “representative” day. The criterion for the selection of the day is based on 8-point HBGM.
2. Four periods of analysis were chosen: baseline (pre-treatment period), mid-treatment period, end-treatment period and follow-up period (post-treatment).
3. For each period the date of HBGM 8-point testing is determined. CGM data for that day is analyzed if there are >50% of the number expected CGM during that day. If the number of CGM determinations on the HBGM 8-point testing day are insufficient then the day before is examined and used if it meets the >50% CGM readings criteria. If that data is insufficient, the same procedure is used for the day after the target date.
4. If none of the three days meet the data sufficiency criteria the subject was dropped from paired MAGE analysis.
5. Only subjects who meet the CGM criteria for the four periods under investigation (pre-treatment, mid-treatment, end-treatment and follow-up) are included.

MAGE calculations:

1. For the selected day determine the standard deviation of all CGM readings.
2. Select the first excursion that exceeds the standard deviation (either exceeds the upper or lower limit of one standard deviation).
3. Determine whether the excursion ascends above or descends below the upper or lower limits of 1 SD. If the first excursion rises above one standard deviation (1SD), then only ascending excursions are used.
4. Identify all excursions that ascend above 1 SD. Measure the difference between the lower and upper limit of each excursion that ascends above 1 SD. Average all of these measurements. This is the MAGE for that day.
5. If, alternatively, the first excursion descends below the lower limit of 1 SD, then all excursions that descend below the lower limit are used, measured and averaged.

Note: Thus, only one direction (ascent or descent) for excursions is employed to calculate MAGE and its determination is based on the direction of the first excursion.
