# Supplementary material for: Diurnal Glycemic Patterns during an 8-Week Open-Label Proof-of-Concept Trial of Empagliflozin in Type 1 Diabetes
Source: PLoS One. 2015 Nov 6;10(11):e0141085. doi: 10.1371/journal.pone.0141085 (PMC4636141; doi:10.1371/journal.pone.0141085)
Supplement: S1 Protocol — (PDF) [file pone.0141085.s004.pdf]

Boehringer Ingelheim

Clinical Trial Protocol

Doc. No.: U11-3038-03

**BI Trial No.:**	1245.46
**BI Investigational Product:**	BI 10773
**Title:**	An open-label 8-week adjunctive-to-insulin and renal mechanistic pilot trial of BI 10773 in type 1 diabetes mellitus (the ATIRMA trial)
**Clinical Phase:**	II
**Trial Clinical Monitor:**	Nima Soleymanlou, Ph.D.<br>The Medical Department<br>Boehringer Ingelheim (Canada) Ltd.<br>5180 South Service Road<br>Burlington, ON L7L 5H4, Canada<br>Phone: (+1) 905-631-4606<br>Fax: (+1) 905-637-4293
**Principal Investigator:**	David Cherney, MD CM, PhD, FRCP(C)<br>Clinician Scientist - Division of Nephrology, University Health Network<br>Assistant Professor of Medicine - University of Toronto<br>Phone: (+1) 416-340-4151<br>Fax: (+1) 416-340-4999
**Status:**	Final Protocol (Revised Protocol (based on global amendment 2))
**Version and Date:**	Version: 3 Date: 20 AUGUST 2012

Proprietary confidential information.
© 2012 Boehringer Ingelheim International GmbH or one or more of its affiliated companies. All rights reserved.
This document may not - in full or in part - be passed on, reproduced, published or otherwise used without prior written permission.

CLINICAL TRIAL PROTOCOL SYNOPSIS

**Name of company:**<br><br>Boehringer Ingelheim<br>**Name of finished product:**<br><br>**Name of active ingredient:**<br><br>BI 10773		**Tabulated Trial Protocol**	
**Protocol date:**<br>**28 Nov 2011**	**Trial number:**<br>1245.46		**Revision date:**<br>**20 Aug 2012**
**Title of trial:**	An open-label 8-week adjunctive-to-insulin and renal mechanistic pilot trial of BI 10773 in type 1 diabetes mellitus (the ATIRMA trial)		
**Principal Investigator:**	David Cherney, MD CM, PhD, FRCP(C)<br>Clinician Scientist - Division of Nephrology, University Health Network<br>Assistant Professor of Medicine - University of Toronto<br>Phone: (+1) 416-340-4151<br>Fax: (+1) 416-340-4999		
**Trial site:**	Single centre study conducted in Canada		
**Clinical phase:**	II		
**Objectives:**	There are two separate objectives in this trial:<br>• The primary objective is to determine the impact of 8 weeks of treatment with BI 10773 (25 mg QD) on renal hyperfiltration in subjects with type 1 diabetes mellitus (T1DM) under conditions of controlled euglycaemia and hyperglycaemia<br>• The secondary objective is to characterize the safety and efficacy of BI 10773 (25 mg QD) in subjects with type 1 diabetes mellitus on insulin pump or multiple daily injections (MDI) therapy		
**Methodology:**	Open-label, single centre mechanistic pilot study to characterize: 1) the impact of BI 10773 on glomerular filtration and 2) the safety and efficacy of BI 10773-treatment as adjunctive-to-insulin therapy in T1DM		
**No. of subjects:**			
**total entered:**	Approximately 30 subjects		
**each treatment:**	All 30 subjects will receive BI 10773 25 mg QD.		
**Diagnosis :**	This mechanistic pilot study will be performed in adult subjects with type 1 diabetes mellitus (T1DM) who are on insulin pump therapy or MDI treatment and have an eGFR $\geq$ 60 ml/min/1.73m^2 at screening		

Proprietary confidential information.
© 2012 Boehringer Ingelheim International GmbH or one or more of its affiliated companies. All rights reserved.
This document may not - in full or in part - be passed on, reproduced, published or otherwise used without prior written permission.

**Name of company:**<br><br>Boehringer Ingelheim	**Tabulated**<br>**Trial Protocol**		
**Name of finished product:**			
**Name of active ingredient:**<br><br>BI 10773			
**Protocol date:**<br>**28 Nov 2011**	**Trial number:**<br>1245.46		**Revision date:**<br>**20 Aug 2012**

**Main criteria for inclusion:**	1. Male or female subject diagnosed with type 1 diabetes mellitus at least 12 months prior to informed consent<br>2. Age ≥ 18 years<br>3. Signed and dated written informed consent by date of Visit 1 in accordance with GCP and local legislation<br>4. HbA_{1C} of 6.5% to 11.0% at Visit 1 (no more than 5 subjects with an HbA_{1C} between 6.5% to <7.0% will be included)<br>5. BMI (Body Mass Index) of 18.5 kg/m^2 to 35.0 kg/m^2 at Visit 1 (Screening)<br>6. Subjects must be either:<br>• experienced insulin pump users (≥ 3 months of use prior to Visit 3 and willing to use the same insulin pump during the course of the study) or<br>• be on multiple daily injections (MDI) of any type of insulin (and be willing to continue on MDI therapy during the course of the study)<br>7. Subject must follow an established and individualized carbohydrate counting method and an insulin titration algorithm based on investigator recommendations<br>8. Subjects must have had a stable glycaemic status defined by their latest HbA_{1C} value as measured 2-12 months prior to screening which must be within 1.5% of the baseline screening value (Visit 1)<br>9. Subjects must have an eGFR ≥ 60 ml/min/1.73m² at screening<br>10. Subject must be able and willing to perform study assessments
**Test product:**	BI 10773
**dose:**	25 mg, once daily
**mode of admin. :**	Tablet per os
**Comparator products:**	A comparator product is not included in this study
**dose:**	This is not applicable per above
**mode of admin. :**	This is not applicable per above
**Duration of treatment:**	Placebo run in period : 2 weeks<br>Treatment period: 8 weeks
**Criteria for efficacy:**	*Primary renal endpoint:*<br>The primary exploratory renal endpoint is the change in glomerular filtration rate (GFR) after treatment with BI 10773 under controlled conditions of euglycaemia and hyperglycaemia.<br><br>*Other exploratory renal endpoints:*<br>The other renal exploratory endpoints after treatment with BI 10773 under controlled

Proprietary confidential information.
© 2012 Boehringer Ingelheim International GmbH or one or more of its affiliated companies. All rights reserved.
This document may not - in full or in part - be passed on, reproduced, published or otherwise used without prior written permission.

**Name of company:**<br><br>Boehringer Ingelheim		**Tabulated Trial Protocol**	
**Name of finished product:**			
**Name of active ingredient:**<br><br>BI 10773			
**Protocol date:**<br>**28 Nov 2011**	**Trial number:**<br>1245.46		**Revision date:**<br>**20 Aug 2012**

conditions of euglycaemia and hyperglycaemia include the following:

- Change in renal hemodynamic function: ERPF, RBF, FF and RVR
- Change in systemic hemodynamic function: MAP and arterial stiffness
- Change in circulating levels of mediators involved in Renin-Angiotensin-Aldosterone System (RAAS) activation and markers of sympathetic activity
- Change in urinary measurements of nitric oxide, prostanoids and albumin excretion

Other exploratory diabetes mellitus endpoints:

The other exploratory diabetes endpoints include:

- Change from baseline in HbA_{1c} after 8 weeks of treatment with BI 10773
- Change from baseline in ~~glycated albumin (GA)~~ **fructosamine (FA)** after 8 weeks of treatment with BI 10773
- Change in fasting plasma glucose from baseline
- Change in glycaemic exposure, variability and stability as derived from Continuous Glucose Monitoring (CGM) from baseline
- Change in parameters of glucose variability (as measured by CGM or home blood glucose monitoring [HBGM])
- Change in mean daily glucose (as derived from the 8-point HBGM)
- Change in AUC for glucose (CGM data) from baseline
- Change in basal, bolus and total insulin requirement from baseline
- Change in basal insulin requirement day vs. night from baseline
- Change in the numbers of bolus boosts from baseline
- Change in UGE from baseline
- Change in BP from baseline
- Change in the Diabetes Treatment Satisfaction Questionnaire from baseline
- Change in HbA_{1c}, hypoglycaemia and insulin requirements from baseline (refer to 7.3.2 for details responder classification)

Proprietary confidential information.
© 2012 Boehringer Ingelheim International GmbH or one or more of its affiliated companies. All rights reserved.
This document may not - in full or in part - be passed on, reproduced, published or otherwise used without prior written permission.

Name of company:<br><br>Boehringer Ingelheim		Tabulated<br>Trial Protocol	
Name of finished product:			
Name of active ingredient:<br><br>BI 10773			
Protocol date:<br>28 Nov 2011	Trial number:<br>1245.46		Revision date:<br>20 Aug 2012
Criteria for safety:	The safety endpoints include the following:<br>• Incidence of adverse events<br>• Protocol-specified significant adverse events<br>• Hypoglycaemic events: Occurrence of serious hypoglycaemia, symptomatic hypoglycaemia (mild or moderate) and time spent in CGM defined hypoglycaemia<br>• Hypoglycaemic events or time spent in CGM-defined hypoglycaemia day vs. night<br>• Change in frequency of asymptomatic hypoglycaemia <3.0 mmol/l (by home blood glucose monitoring) from baseline<br>• Change in weight from baseline<br>• Change in waist circumference<br>• Changes from baseline in clinical laboratory values (e.g. electrolytes)<br>• Vital signs		
Statistical methods:	<u>Primary analysis:</u><br>Paired t-tests will be performed to test the difference in the GFR response between baseline and after treatment of BI 10773 (25 mg QD) in hyperfilterer subjects under separate conditions of controlled euglycaemia and hyperglycaemia.<br><br><u>Secondary analysis:</u><br>The secondary analyses will focus on additional between-group and within-group comparisons of the primary endpoint.<br><br><u>Other exploratory renal analyses:</u><br>For the other exploratory renal endpoints, between-group and within-group comparisons will be performed.<br><br><u>Other exploratory diabetes analyses:</u><br>Descriptive statistics will be used to summarize and evaluate the other diabetes-related endpoints.<br><br><u>Safety:</u><br>Descriptive statistics will be used to summarize and evaluate the safety endpoints.		

Proprietary confidential information.
© 2012 Boehringer Ingelheim International GmbH or one or more of its affiliated companies. All rights reserved.
This document may not - in full or in part - be passed on, reproduced, published or otherwise used without prior written permission.

FLOW CHART

Trial Period	Screening	Placebo run-in	Treatment Period (BI 10773 treatment starting at Day 3)												Follow Up	
**Visit**	1	2[A]	3	4	5	6	7	8	9	10	11[A]	12	13	EOT[A]	14	15
**Study week**	-3	-2	0		1	2	3	4	5	6	7	8		Only for early discon-tinued subjects	9	10
**Days from Study day 1 allowed visit window**	-21	-14 -6	1	2	8 ±2	15 ±2	22 ±2	29 ±4	36 ±2	43 ±2	50 ±2	57 ±4	V12 + 1		65 ±2	72 ±2
In-person clinic visit	X	X	X	X				X			X	X	X	X	X	X
Weekly Telephone call					X	X	X		X	X						
Daily Telephone call[B]		X[B]		X[B]												
Informed Consent	X															
Inclusion/exclusion criteria	X	X	X	X												
Clamped euglycaemia			X[C]									X[D]				
Clamped hyperglycaemia				X[C]									X[D]			
SNS / arterial stiffness			X	X								X	X			
Medical History/Demographics	X															
Physical exam	X							X					X	X		
Vital signs	X	X	X	X				X				X	X	X		X
Height	X															
Weight	X		X					X				X		X		X
Waist circumference			X					X				X		X		X
12-lead-ECG[E]	X												X	X		
Diet & exercise counselling[F]		X[G]	X	X	X	X	X	X	X	X	X[G]	X	X	X	X	X
Pregnancy Test[H]	X												X	X		
Daily FPG via HBGM[I]		X	X	X	X	X	X	X	X	X	X	X	X	X	X	X
8-point HBGM profile[J]		X					X			X					X	
CGM[B]		X	X	X	X	X	X	X	X	X	X	X	X	X	X	X
Blood and Urine[K]	X	X	X	X				X			X	X	X	X		X[K]
Pre-dose PK Sample[K]								X			X	X				
24-hour urine collection[L]		X									X					
Glycated hemoglobin (HbA_{1c})	X	X	X					X			X	X		X		X
~~Glycated Albumin (GA)~~ **Fructosamine (FA)**		X	X					X			X	X		X		X
Fasting Plasma Glucose (FPG)		X									X			X		
Adverse events		X	X	X	X	X	X	X	X	X	X	X	X	X	X	X
DTSQs[M]			X					X				X				
Concomitant Therapy	X	X	X	X	X	X	X	X	X	X	X	X	X	X	X	X
Dispense placebo run-in[N]		X														
Dispense BI 10773 25 mg[O]				X				X								
Medication compliance			X		X[P]	X[P]	X[P]	X	X[P]	X[P]	X	X	X	X		
Trial termination																X

Proprietary confidential information.
© 2012 Boehringer Ingelheim International GmbH or one or more of its affiliated companies. All rights reserved.
This document may not - in full or in part - be passed on, reproduced, published or otherwise used without prior written permission.

A. Visits 2, 11 and if required EOT are the only clinic visits where a fasting status is required for labs. Visit 2 to be performed preferably within 1 week of Visit 1. If subjects discontinue the trial after the start of therapy and prior to completion of Visit 13, an end of treatment (EOT) visit should be done as soon as possible and the following will be performed: A physical exam including vitals, an ECG and laboratory assessments (FPG, HbA_{1c}, ~~GA~~ **FA**, Hematology, Chemistry, Lipids, Urine B and hCG). The EOT visit must be performed instead of a scheduled clinic visit for early discontinued subjects in cases where discontinuation occurs during a scheduled clinic visit. Early discontinued subjects should return to the clinic only for follow up Visit 15 (Visit 14 may be skipped). Refer to Section 6.2.3 for details. If feasible, early discontinued subjects should continue with CGM and HBGM until Visit 15.

B. During the run-in period, subjects should be contacted preferably daily or every 3-6 days to ensure that they are properly calibrating the Continuous Glucose Monitoring (CGM) device (daily) and replacing the CGM sensor as required (sensor change required after 6 days of use). After the start of treatment with BI 10773, subjects should be preferably contacted daily from study Day 3 to Day 7 to ensure that study compliance and safety is achieved. Subjects will be expected to use CGM until Visit 15. Details of subject follow-ups during the run-in period and during the study Days 3-7 will be captured in eCRFs.

C. Baseline assessment of renal hemodynamic function at the renal physiology laboratory:

Time	Assessments under euglycaemic clamp (Day 1) and hyperglycaemic clamp (Day 2):
07:45	Admittance to laboratory and preparation for clamp procedures to maintain desired glycaemia (4-6 mmol/l during Day 1 and 9-11 mmol/l during Day 2) throughout assessments for that day
12:00	Start inulin and PAH infusion
13:30-14:00	Continued Inulin and PAH infusion, assessment of MAP and clearance measurements (GFR, ERPF, FF, RBF, RVR) each based on the average of 2 baseline measurements, baseline RAAS mediators, NO, prostanoid measurements, arterial stiffness and markers of sympathetic nervous system activation (heart rate variability and circulating plasma levels of adrenaline/noradrenaline) and other laboratory assessments.

D. Assessment of renal hemodynamic function at the renal physiology laboratory after 8 weeks of BI 10773 treatment:

Time	Assessments under euglycaemic clamp (Day 57) and hyperglycaemic clamp (Day 58):
07:45	Admittance to laboratory and preparation for clamp procedures to maintain desired glycaemia (4-6 mmol/l during Day 57 and 9-11 mmol/l during Day 58) throughout assessments. A pre-dose PK sample is collected during study Day 57 at **approximately** the same time as PK collections at Visits 8 and 11. Refer to footnote N for the time of drug dosing during Days 57 and 58.
12:00	Administration of BI 10773 25 mg and start inulin and PAH infusion
13:30-14:00	Repeat measurements of MAP, GFR, ERPF, FF, RBF and RVR (all average of 2 measurements), RAAS mediators, NO, prostanoids, arterial stiffness and markers of sympathetic nervous system activation (heart rate variability and circulating plasma levels of adrenaline/noradrenaline) and other laboratory assessments.

E. In addition to the required ECG at Visit 1 and Visit 13, additional ECGs should be performed in cases of cardiac symptoms (rhythm disorders or cardiac ischemia).

F. Subjects will be advised to follow a healthy diet and exercise program based on investigator recommendations during the study except during the 7 days preceding the start of renal physiology laboratory assessments when they will be instructed to follow a modified diet, refer to G below for details.

G. Subject will be instructed to adhere to a high-sodium (preferably > 140 mmol/day) and moderate-protein (<1.5g/kg/day) diet during the 7 days preceding the start of renal assessments at baseline during Visits 3 and 4 (the diet to continue until the end of renal assessments at baseline, Visit 4) and during the 7 days preceding the start of the end of treatment renal assessments during Visits 12 and 13 (the diet to continue until the end of renal assessments). The initial 8-point HBGM must be performed prior to start of the modified diet (i.e. prior to day -7). If a subject fails to adhere to the prescribed high-sodium and moderate-protein diet, the investigator will assess whether the subject is still eligible to start the renal assessments based on the subject's actual diet during the 7 days preceding Visit 3. In cases of an unsatisfactory diet, subjects may have to be screen failed based on the investigator's judgement.

Proprietary confidential information.
© 2012 Boehringer Ingelheim International GmbH or one or more of its affiliated companies. All rights reserved.
This document may not - in full or in part - be passed on, reproduced, published or otherwise used without prior written permission.

H. A local pregnancy test will be done in women of child bearing potential. More frequent testing can be done if required by the local regulation and/or authority or per investigator judgement.

I. During the screening, treatment and follow up periods, several daily measurements of plasma glucose are recommended including a fasted daily measurement and additional daily measurements including before and after meals and at bedtime. Unscheduled measurements must always be done in cases of hypo- or hyperglycaemia-related symptoms. All HBGM results will be captured in a subject study diary. *Treatment decisions must always be based on blood glucose values as determined by using a home blood glucose meter.*

J. Four specific periods of 8-point HBGM will be conducted and results will be recorded by the subject at home in a specific HBGM 8-point log. The 8-point weighted mean daily glucose (MDG) profile must be performed *1 day after Visit 2 and Visit 14 and one day prior to Visits 8 and 11 (thus subjects should start the 8-point test in the mornings of study days -13, 28, 49 and 66).* The 8 points of measurements will be: (#1) after a 10 hour fast before study drug intake and 0-5 mins prior to intake of breakfast , (#2) 90 mins after breakfast, (#3) 0-5 mins prior to intake of lunch, (#4) 90 mins after lunch, (#5) 0-5 mins prior to intake of dinner, (#6) 90 mins after dinner, (#7) 0-5 mins prior to bedtime and (#8) after a 10 hour fast before study drug intake and 0-5 mins prior to intake of breakfast (the next day).

K. Collection of safety / efficacy labs and PK samples will be done based on the attached table, also listed in Section 5.2.3. For Visit 15, if required, other panels/tests may be repeated (fasted measurements as needed) based on clinically significant findings at Visits 11-13 or per investigator judgment.

Visit 1	*Visit 2*	Visit 3	Visit 4	Visit 8	*Visit 11*	Visit 12	Visit 13	*(EOT)*	Visit 15
Day -21	Day -14	Day 1	Day 2	Day 29	Day 50	Day 57	Day 58	Only for early discontinued subjects (after start of therapy)	Day 72
Non-Fasted	*Fasted*	Non-Fasted	Non-Fasted	Non-Fasted	*Fasted*	Non-Fasted	Non-Fasted	*Fasted*	Non-Fasted
HbA_{1c}<br>Hematology<br>Chemistry<br>TSH<br>Urine A/B<br>Urine: albumin and creatinine<br>Serum hCG	FPG<br>HbA_{1c}<br>~~GA~~ **FA**<br>Hematology<br>Chemistry<br>Lipids<br>Urine A/B	HbA_{1c}<br>~~GA~~ **FA**<br>Hematology<br>Chemistry<br>Renal panel<br>Urine A/B/C	Hematology<br>Chemistry<br>Renal panel<br>Urine A/B/C	HbA_{1c}<br>~~GA~~ **FA**<br>Hematology<br>Chemistry<br>Urine A/B	FPG<br>HbA_{1c}<br>~~GA~~ **FA**<br>Hematology<br>Chemistry<br>Lipids<br>Urine A/B	HbA_{1c}<br>~~GA~~ **FA**<br>Hematology<br>Chemistry<br>Renal panel<br>Urine A/B/C	Hematology<br>Chemistry<br>Renal panel<br>Urine A/B/C<br>Serum hCG	FPG<br>HbA_{1c}<br>~~GA~~ **FA**<br>Hematology<br>Chemistry<br>Lipids<br>Urine B<br>Serum hCG	HbA_{1c}<br>~~GA~~ **FA**
				Pre-dose PK	Pre-dose PK (same time as V8)	Pre-dose PK (same time as V8)			

L. To demonstrate the desired sodium excretion level (preferably > 140 mmol/day) following the prescribed high-sodium / moderate-protein diet, the site is to remind the subject to perform a 24-hour urine collection on study Days -1 (collection period from day-1 to day 1) and 56 (collection period from day 56 to 57), hence the collection of urine to start the day before clamped procedures are performed and to end the morning of Visit 3 and Visit 12. It is expected that subjects would achieve a urinary sodium excretion rate > 140 mmol/day after following the prescribed high-sodium / moderate-protein diet for 7 days. Daily diet compliance will be captured by the subject in study-specific logs. In addition to sodium; glucose, urea and creatinine will also be measured in the 24-hour urine sample.

M. For treatment satisfaction, the Diabetes Treatment Satisfaction Questionnaire will be used in its status version (DTSQs) and data will be collected in eCRFs.

N. Subjects should start taking the placebo run-in medication in the morning (7-11 AM) of the day following Visit 2 and continue daily intake until Visit 3. Subjects must bring to the clinic Visit 3 all previously dispensed medication and packaging.

Proprietary confidential information.
© 2012 Boehringer Ingelheim International GmbH or one or more of its affiliated companies. All rights reserved.
This document may not - in full or in part - be passed on, reproduced, published or otherwise used without prior written permission.

O. The trial medication (BI 10773) will be dispensed at the end of Visit 4 (study Day 2). Subject will be instructed to start taking the trial medication (BI 10773 25 mg tablet once daily) as of the morning of study Day 3. Subjects should continue to take the study drug daily at the same time every day (7-11 AM). *Subject will be reminded not to take the trial medication the morning of clinic Visits 8, 11, 12 and 13.* During study Visits 12 and 13, BI 10773 dosing will be done at the clinic approximately 1.5 hours before the start of the planned renal assessments at around 12:00 (refer to footnote D for details). The PK samples for Visits 11 and 12 should be collected at **approximately** the same time as Visit 8.

P. Subjects will be reminded during telephone Visits 5, 6, 7, 9 and 10 to take their medication once daily in the morning between 7-11 AM. The medication compliance check will be performed during clinic Visits 3 (for placebo run-in) and visits 8, 11, 12 and 13 (for BI 10773) when subjects will bring to the clinic all previously dispensed medication and packaging.

Proprietary confidential information.
© 2012 Boehringer Ingelheim International GmbH or one or more of its affiliated companies. All rights reserved.
This document may not - in full or in part - be passed on, reproduced, published or otherwise used without prior written permission.

TABLE OF CONTENTS

TITLE PAGE 1
CLINICAL TRIAL PROTOCOL SYNOPSIS 2
FLOW CHART 6
TABLE OF CONTENTS 10
ABBREVIATIONS 13
1. INTRODUCTION 16
1.1 MEDICAL BACKGROUND 16
1.2 DRUG PROFILE 18
2. RATIONALE, OBJECTIVES, AND BENEFIT - RISK ASSESSMENT 20
2.1 RATIONALE FOR PERFORMING THE TRIAL 20
2.2 TRIAL OBJECTIVES 20
2.3 BENEFIT - RISK ASSESSMENT 20
3. DESCRIPTION OF DESIGN AND TRIAL POPULATION 24
3.1 OVERALL TRIAL DESIGN AND PLAN 24
3.1.1 Administrative structure of the trial 24
3.2 DISCUSSION OF TRIAL DESIGN, INCLUDING THE CHOICE OF CONTROL GROUP 25
3.3 SELECTION OF TRIAL POPULATION 26
3.3.1 Main diagnosis for study entry 26
3.3.2 Inclusion criteria 27
3.3.3 Exclusion criteria 27
3.3.4 Removal of subjects from therapy or assessments 29
3.3.4.1 Removal of individual subjects 29
3.3.4.2 Discontinuation of the trial by the sponsor 30
4. TREATMENTS 31
4.1 TREATMENTS TO BE ADMINISTERED 31
4.1.1 Identity of BI investigational product and comparator product 31
4.1.2 Method of assigning subjects to treatment groups 31
4.1.3 Selection of doses in the trial 32
4.1.4 Drug assignment and administration of doses for each subject 32
4.1.5 Blinding and procedures for unblinding 33
4.1.5.1 Blinding 33
4.1.5.2 Procedures for emergency unblinding 33
4.1.6 Packaging, labelling, and re-supply 33
4.1.7 Storage conditions 33
4.1.8 Drug accountability 34
4.2 CONCOMITANT THERAPY, RESTRICTIONS, AND RESCUE TREATMENT 34
4.2.1 Rescue medication, emergency procedures and additional treatments 35
4.2.2 Restrictions 35

Proprietary confidential information.
© 2012 Boehringer Ingelheim International GmbH or one or more of its affiliated companies. All rights reserved.
This document may not - in full or in part - be passed on, reproduced, published or otherwise used without prior written permission.

4.2.2.1 Restrictions regarding concomitant treatment....35
4.2.2.2 Restrictions on diet and life style....36
4.3 TREATMENT COMPLIANCE....36
5. VARIABLES AND THEIR ASSESSMENT....37
5.1 EFFICACY - PHARMACODYNAMICS....37
5.1.1 Endpoints of efficacy....37
5.1.2 Assessment of efficacy....38
5.1.2.1 Hemodynamic measurements....38
5.1.2.2 Arterial stiffness and sympathetic nervous system (SNS) measurements....38
5.1.2.3 Blood pressure and pulse rate....39
5.1.2.4 Fasting plasma glucose....39
5.1.2.5 Glycated hemoglobin (HbA_{1c}) and ~~glycated albumin (GA)~~ **Fructosamine (FA)**....39
5.2 SAFETY....39
5.2.1 Endpoints of safety....39
5.2.2 Assessment of adverse events....40
5.2.2.1 Definitions of adverse events....40
5.2.2.2 Adverse event and serious adverse event reporting....42
5.2.3 Assessment of safety and efficacy laboratory parameters....43
5.2.4 Physical examination and electrocardiogram....48
5.2.5 Weight and waist circumference....48
5.3 OTHER....49
5.3.1 Other endpoint....49
5.3.1.1 The Diabetes Treatment Satisfaction Questionnaire (DTSQ)....49
5.3.2 Other assessments....49
5.3.2.1 Home Blood Glucose Monitoring (HBGM)....49
5.3.2.2 Continuous Glucose Monitoring (CGM) Evaluation....50
5.3.3 Pharmacogenomic evaluation....51
5.4 APPROPRIATENESS OF MEASUREMENTS....51
5.5 DRUG CONCENTRATION MEASUREMENTS AND PHARMACOKINETICS....51
5.5.1 Pharmacokinetic endpoints....51
5.5.2 Methods of sample collection....51
5.5.3 Analytical determinations....52
5.6 BIOMARKERS....52
5.6.1 Endpoints based on biomarkers....52
5.6.2 Methods of sample collection....52
5.6.3 Analytical determinations....52
5.7 PHARMACODYNAMICS....52
5.7.1 Pharmacodynamic endpoints....52
5.7.2 Methods of sample collection....52
5.8 PHARMACOKINETIC - PHARMACODYNAMIC RELATIONSHIP....52
6. INVESTIGATIONAL PLAN....53
6.1 VISIT SCHEDULE....53

Proprietary confidential information.
© 2012 Boehringer Ingelheim International GmbH or one or more of its affiliated companies. All rights reserved.
This document may not - in full or in part - be passed on, reproduced, published or otherwise used without prior written permission.

6.2 DETAILS OF TRIAL PROCEDURES AT SELECTED VISITS.................53
6.2.1 Screening and run-in period.................53
6.2.2 Treatment period.................54
6.2.3 End of trial and follow-up period.................56
7. STATISTICAL METHODS AND DETERMINATION OF SAMPLE SIZE.................57
7.1 STATISTICAL DESIGN - MODEL.................57
7.2 NULL AND ALTERNATIVE HYPOTHESES.................58
7.3 PLANNED ANALYSES.................59
7.3.1 Primary analysis.................59
7.3.2 Secondary analyses.................60
7.3.2.1 Secondary analyses.................60
7.3.2.2 Other analyses.................60
7.3.3 Safety analyses.................63
7.3.4 Interim analyses.................64
7.3.5 Pharmacokinetic analyses.................64
7.3.6 Pharmacodynamic analyses.................64
7.3.7 Pharmacogenomic analyses.................64
7.4 HANDLING OF MISSING DATA.................64
7.5 RANDOMISATION.................64
7.6 DETERMINATION OF SAMPLE SIZE.................64
8. INFORMED CONSENT, DATA PROTECTION, TRIAL RECORDS.................66
8.1 STUDY APPROVAL, PATIENT INFORMATION, AND INFORMED CONSENT.................66
8.2 DATA QUALITY ASSURANCE.................67
8.3 RECORDS.................67
8.3.1 Source documents.................67
8.3.2 Direct access to source data and documents.................67
8.4 LISTEDNESS AND EXPEDITED REPORTING OF ADVERSE EVENTS.................67
8.4.1 Listedness.................67
8.4.2 Expedited reporting to health authorities and IECs/IRBs.................68
8.5 STATEMENT OF CONFIDENTIALITY.................68
9. REFERENCES.................69
9.1 PUBLISHED REFERENCES.................69
9.2 UNPUBLISHED REFERENCES.................70
10. APPENDICES.................71
10.1 DTSQs.................71
11. DESCRIPTION OF GLOBAL AMENDMENTS.................72

Proprietary confidential information.
© 2012 Boehringer Ingelheim International GmbH or one or more of its affiliated companies. All rights reserved.
This document may not - in full or in part - be passed on, reproduced, published or otherwise used without prior written permission.

ABBREVIATIONS

AE	Adverse Event
AGP	Ambulatory Glucose Profile
AIx	Radial augmentation index
ALT	Alanine aminotransaminase
AP	Alkaline Phosphatase
AST	Aspartate aminotransaminase
AUC	Area Under the Curve
BG	Blood Glucose
BI	Boehringer Ingelheim
BMI	Body Mass Index
BP	Blood Pressure
CGM	Continuous Glucose Monitoring
CI	Confidence Interval
CML	Local Clinical Monitor
CRA	Clinical Research Associate
CRF	Case Report Form
CSII	Continuous Subcutaneous Insulin Infusion
CTCAE	Common Terminology Criteria for Adverse Events
CTMF	Clinical Trial Master File
CTP	Clinical Trial Protocol
CTR	Clinical Trial Report
DBP	Diastolic Blood Pressure
dL	Decilitre
DMC	Data-Monitoring Committee
DTSQ	Diabetes Treatment Satisfaction Questionnaire
ECG	Electrocardiogram
eCRF	Electronic Case Report Form
eGFR	Estimated Glomerular Filtration Rate
EDC	Electronic Data Capture
EDTA	Ethylenediaminetetraacetic acid
EOT	End Of Treatment
ERPF	Effective Renal Plasma Flow
ESRD	End Stage Renal Disease
EudraCT	European Clinical Trials Database
**FA**	**Fructosamine**
FAS	Full Analysis Set
FF	Filtration Fraction
FPG	Fasting Plasma Glucose
~~GA~~	~~Glycated Albumin~~
GCP	Good Clinical Practice
GFR	Glomerular Filtration Rate
GTI	Genital Tract Infection
HbA_{1c}	Hemoglobin A1c (glycated hemoglobin)
HBGM	Home Blood Glucose Monitoring

Proprietary confidential information.
© 2012 Boehringer Ingelheim International GmbH or one or more of its affiliated companies. All rights reserved.
This document may not - in full or in part - be passed on, reproduced, published or otherwise used without prior written permission.

hCG	Human Chorionic Gonadotropin
HPLC	High-Performance Liquid Chromatography
HPC	Human Pharmacology Centre
hr	Hour
IB	Investigator's Brochure
IEC	Independent Ethics Committee
IRB	Institutional Review Board
ISF	Investigator Site File
IU	International Unit
i.v.	intravenous
Kg	Kilogram
LTs	Liver Transaminases
m	Meter
MAP	Mean Arterial Pressure
MAGE	Mean Amplitude of Glycaemic Excursions
MDI	Multiple Daily Injections
MDG	Mean Daily Glucose
MedDRA	Medical Dictionary for Drug Regulatory Activities
mg	Milligram
min	Minute
ml	Millilitre
mmol	Millimole
MS	Mass Spectrometry
MST	Medical Subteam
NO	Nitric Oxide
NOAEL	No Observed Adverse Effect Level
OPU	Operative Unit
p.o.	per os (oral)
PAH	Paraaminohippurate
PCC	Protocol Challenge Committee
PK	Pharmacokinetic
PWV	Pulse Wave Velocity
q.d. (QD)	quaque die (once a day)
RAAS	Renin-Angiotensin-Aldosterone System
RBF	Renal Blood Flow
RVR	Renal Vascular Resistance
SAE	Serious Adverse Event
SBP	Systolic Blood Pressure
s.c.	Subcutaneous
SD	Standard Deviation
SGLT-2	Sodium-dependent Glucose co-Transporter 2
SLC5	Sodium-Glucose Co-transport 5
SNS	Sympathetic Nervous System
SOP	Standard Operating Precedure
T1DM	Type 1 Diabetes Mellitus
T2DM	Type 2 Diabetes Mellitus

Proprietary confidential information.
© 2012 Boehringer Ingelheim International GmbH or one or more of its affiliated companies. All rights reserved.
This document may not - in full or in part - be passed on, reproduced, published or otherwise used without prior written permission.

TCM	Trial Clinical Monitor
TGF	Tubuloglomerular Feedback
TSAP	Trial Statistical Analysis Plan
TSH	Thyroid Stimulating Hormone
UGE	Urinary Glucose Excretion
USA	United States of America
UTI	Urinary Tract Infections
V	Visit
WHO	World Health Organization

Proprietary confidential information.
© 2012 Boehringer Ingelheim International GmbH or one or more of its affiliated companies. All rights reserved.
This document may not - in full or in part - be passed on, reproduced, published or otherwise used without prior written permission.

1. INTRODUCTION

BI 10773 is an orally available inhibitor of the sodium-glucose co-transporter 2 (SGLT-2), which promotes enhanced glucose excretion in the urine, thereby lowering blood glucose concentrations. It is currently being evaluated in early phase III trials in subjects with type 2 diabetes mellitus (T2DM), but this compound, due to its non-insulin dependent mode of action also has the potential to lower blood glucose concentrations in subjects with type 1 diabetes mellitus (T1DM).

1.1 MEDICAL BACKGROUND

T1DM accounts for 5 to 10% of all cases of diabetes. This disease, a complex disorder that requires constant attention to diet, exercise, glucose monitoring, and insulin therapy to achieve good glycaemic control, occurs as a consequence of the organ-specific immune destruction of the pancreas' insulin-producing β-cells in the islets of Langerhans (R10-6370).

Maintaining HbA_{1c} levels < 7.0% in individuals with T1DM has been shown to reduce macrovascular and microvascular complications. Despite advances in insulin formulation and delivery, such as the development of continuous subcutaneous insulin infusion (CSII) systems, continuous glucose monitoring (CGM) systems and refinement of pharmacokinetic properties of rapid- and long-acting insulin analogues, current therapy for subjects with diabetes requiring insulin often does not lead to satisfactory glycaemic control. In fact, only a minor portion of subjects achieve normalization of HbA_{1c} and restoration of euglycaemia. Most subjects achieve HbA_{1c} levels generally not lower than 8.0 %. Hence, with the currently available treatment options, subjects with T1DM fail to maintain adequate blood glucose control. This does not only lead to acute conditions such as severe hypoglycaemia and ketoacidosis but also to debilitating secondary complications including heart disease, blindness and kidney failure (R10-6370, R10-6371).

Reaching HbA_{1c} goals by intensifying the insulin regimen is accompanied by increased incidence of hypoglycaemic events. The Diabetes Control and Complications Trial and the Oxford- Steno group population study have demonstrated that severe hypoglycaemia affects about one third of subjects with T1DM. Therefore, the occurrence of hypoglycaemia hinders the efficacy of intensive insulin therapy in subjects with diabetes (R10-6369).

Moreover, data on self-reported insulin use suggests that subjects frequently omit injections for various reasons. In 31% of women who reported intentionally omitting doses (8% frequently), weight gain was the reason. One-fourth of adolescents reported having omitted some injections during the 10 days before a clinic visit. Therefore, clinicians cannot assume that subjects with T1DM are fully compliant with insulin regimens (R10-6376). The injurious effects of improper glycaemic control in such cases will in the long run result in macrovascular and microvascular complications.

With respect to macrovascular problems, the Finnish Diabetic Nephropathy study (FinnDiane) has demonstrated that the overall prevalence of metabolic syndrome in subjects with T1DM (39%) is approximately three times that observed in non-diabetic subjects in the general Finnish population but lower than the incidence observed in subjects with T2DM

Proprietary confidential information.
© 2012 Boehringer Ingelheim International GmbH or one or more of its affiliated companies. All rights reserved.
This document may not - in full or in part - be passed on, reproduced, published or otherwise used without prior written permission.

(78–84%). In particular, more than 50% of subjects with poor glycaemic control or renal impairment fulfilled the National Cholesterol Education Program (NCEP) diagnostic criteria for metabolic syndrome. In addition, approximately 33% of subjects without nephropathy or with good glycaemic control met the diagnostic criteria (R10-6377). Given the strong association between components of the metabolic syndrome (including dyslipidemia and hypertension) and vascular complications, subjects with T1DM who meet the criteria for metabolic syndrome may be at the greatest risk of macrovascular complications.

With respect to microvascular complications, it is estimated that 40% of subjects with end stage renal disease (ESRD) requiring renal replacement therapy have an underlying diagnosis of nephropathy due to diabetes, and this rate is increasing annually (R10-6368). The cost of dialysis presents a significant burden to healthcare systems of industrialized nations. The costs of managing ESRD in Canada alone are approximately $50,000.00 per subject per year or nearly 1 billion/year and increasing.

As such, the need to develop a therapy with the potential to improve glycaemic control in subjects with T1DM and to curb the progression of diabetes-associated renal complications is urgently warranted. Such a therapy could significantly decrease the healthcare costs associated with glycaemic control regimens, pre-dialysis care and renal replacement therapies.

In this study, BI 10773, an oral SGLT-2 inhibitor, will be studied in T1DM as an adjunct to insulin treatment. SGLT-2 is a member of a larger group of sodium substrate co-transporters, the sodium-glucose co-transport 5 (SLC5) gene family (R05-0939). The pharmacological rationale to administer an SGLT-2 inhibitor in T1DM is based on the fact that the kidney plays a pivotal role in the regulation of the plasma glucose concentration. The kidney, as part of one of its major roles, reabsorbs the glucose filtered by the glomeruli (~ 162 grams/day of glucose in healthy subjects based on approximately 180 litres of plasma filtered multiplied by an average glucose concentration of approximately 90 mg/dl). In normal glucose-tolerant subjects, virtually all of this filtered glucose is reabsorbed in the proximal tubule of nephrons. The SGLT-2 protein is almost exclusively expressed in the renal proximal tubules and accounts for 90% of the renal glucose reabsorption in healthy subjects (the remaining 10% are accounted for by another co-transporter, the SGLT-1). Because sodium is transported along its electrochemical gradient, it provides the energy required for the SGLT to transport glucose against its concentration gradient into the renal proximal tubular cell. The sugar exits the basolateral cell border via the GLUT2 transporter (a glucose transporter that passively facilitates glucose movement along its chemical gradient).

It was demonstrated, nearly four decades ago, that in subjects with poorly controlled T1DM, the maximum tubular reabsorption capacity for glucose was significantly increased (R10-6572). Interestingly, one would in fact have suspected that during hyperglycaemia, with increased interstitial and intracellular glucose concentrations, the reduced glucose concentration gradient across the basolateral membrane of the proximal renal epithelial cell would attenuate glucose efflux. This observation could be explained by the fact that tubular cells cultured from subjects with T2DM have up-regulated glucose transporters, including SGLT-2. In many respects, this phenomenon (i.e., the increased reabsorption of glucose when

Proprietary confidential information.
© 2012 Boehringer Ingelheim International GmbH or one or more of its affiliated companies. All rights reserved.
This document may not - in full or in part - be passed on, reproduced, published or otherwise used without prior written permission.

glucose concentration is elevated) could therefore be considered a maladapted response to glucosuria in diabetes, since it would rather be desirable for the kidney to excrete the excess filtered glucose load in order to restore normoglycaemia (R10-0703).

Hence, based on these pathophysiological considerations, BI 10773, as an oral SGLT-2 inhibitor, has the potential to provide a novel approach to the treatment of T1DM, as adjunctive therapy to insulin. BI 10773 lowers both the saturation threshold and the maximal transport capacity of SGLT-2 for glucose, resulting in increased glucosuria, insulin-independent reduction of plasma glucose levels with a potential to reduce the risk of hypoglycaemia and a potential to lower weight in T1DM, due to a negative energy balance. Furthermore, by lowering plasma glucose levels and possibly increasing the sodium load in the distal renal tubular system, SGLT-2 inhibitors may reduce systemic and local RAAS activation leading to blood pressure reduction.

1.2 DRUG PROFILE

Non-clinical assessment of safety

A comprehensive package of safety pharmacology, genetic toxicology, reproductive toxicology and general toxicology studies were conducted in mice, rats, rabbits and dogs to support the chronic administration of BI 10773 to humans. The compound is well tolerated in animals at clinically relevant plasma exposures, while adverse effects were observed at higher exposures. Noteworthy adverse findings at effect levels above the NOAEL (no observed adverse effect level) in general toxicology studies are body weight loss, lower weight gain, dehydration, nephritis and nephropathy. Human clinical exposure at 25 mg/day is well below the exposure at the NOAEL of 100 mg/kg/day in male rats after 26 weeks of and the NOAEL of 10 mg/kg/day in the dog after 52 weeks of dosing and indicates a 9 to 10 fold therapeutic window to these NOAELs. These toxicology data suggest BI 10773 can be safely administered to humans at 50 mg/day in long term studies; refer to the Investigator's Brochure for additional details.

Clinical pharmacokinetics

In humans, BI 10773 highly and selectively blocks glucose transport via SGLT-2 (IC50 1.3 nmol/l), with a 5000-fold selectivity over SGLT-1 (IC50 6278 nM). Refer to the Investigator's Brochure for additional details. BI 10773 is further characterized with a predominantly linear pharmacokinetics following single oral doses and at steady-state after multiple oral doses. BI 10773 was rapidly absorbed reaching peak levels at approximately 1.5 h and showed a biphasic decline with the terminal elimination half-life ranging from 10 to 19 h.

BI 10773 exposure increases moderately with the extent of renal impairment suggesting that a dose adjustment is not required in subjects with renal impairment. The observations from the phase I study in subjects with renal impairment indicate a rather low efficacy of BI 10773 in subjects with severe renal impairment and end stage renal disease (ESRD).

Proprietary confidential information.
© 2012 Boehringer Ingelheim International GmbH or one or more of its affiliated companies. All rights reserved.
This document may not - in full or in part - be passed on, reproduced, published or otherwise used without prior written permission.

Clinical efficacy and safety

More than 190 healthy volunteers were exposed to BI 10773 (dose range: 0.5 mg to 800 mg per single dose and up to 50 mg in multiple dosing). Approximately 170 Caucasian and Japanese subjects with T2DM have been exposed to BI 10773 in phase I studies with a duration of 2-4 weeks and approximately 600 subjects with T2DM have completed up to 12 weeks of treatment with different doses of BI 10773 in two phase II studies; refer to the Investigator's Brochure for additional details.

Increased urinary glucose excretion (UGE) compared with placebo was observed at all doses of BI 10773 in both healthy volunteers and T2DM subjects. The 12-week phase II studies demonstrated an HbA1c reduction of up to 0.72 % (placebo subtracted), a fasting plasma glucose (FPG) reduction of up to 32 mg/dL and a weight loss of approximately 1.5 kg, in both the monotherapy setting and as an add-on to metformin (≥ 1500 mg/day).

Treatment with BI 10773 resulted in a similar percentage of overall Adverse Events (AEs) (approximately 44 %) and Serious Adverse Events (SAEs) (approximately 2%) compared to placebo and/or active comparators. Treatment with BI 10773 showed a higher frequency of genital tract infections and symptoms of pollakiuria/polyuria/nocturia, yet was not associated with a higher incidence of urinary tract infections or hypoglycaemia.

In summary, BI 10773 was well tolerated in Phase I and Phase II studies in healthy male volunteers and subjects with T2DM. The safety profile of BI 10773 was comparable to placebo and there have been no deaths related to BI 10773. The vast majority of AEs considered related to BI 10773 have been of mild to moderate nature. Given the good safety profile in the preclinical studies of BI 10773 at all dose levels and the good tolerability seen in the clinical study program to date, the available clinical and non-clinical data support the further development of BI 10773 in larger studies with long term treatment durations of 26 weeks and above.

For further details refer to the current version of the BI 10773 Investigator's Brochure.

Proprietary confidential information.
© 2012 Boehringer Ingelheim International GmbH or one or more of its affiliated companies. All rights reserved.
This document may not - in full or in part - be passed on, reproduced, published or otherwise used without prior written permission.

2. RATIONALE, OBJECTIVES, AND BENEFIT - RISK ASSESSMENT

2.1 RATIONALE FOR PERFORMING THE TRIAL

Subjects with T1DM and subjects with T2DM are both at a greater risk of developing nephropathy, potentially leading to end stage renal disease. The pathophysiology of these complications may be partly mediated by hyperglycaemia, which results in activation of the renin-angiotensin-aldosterone system (RAAS) leading to renal hemodynamic abnormalities including efferent arteriolar vasoconstriction, an event which is postulated to result in renal hyperfiltration ultimately culminating in organ injury (R10-6368, R10-6364, R10-6365, R10-6366, R10-6367, R10-6375, R10-6374). Modulation of the renal tubuloglomerular feedback, via the direct pharmacologic inhibition of the SGLT-2, may re-establish normal kidney filtration in hyperfilterer subjects and thus over time, slowdown kidney injury.

Blockade of sodium reabsorption at the proximal tubule of nephrons is likely to increase sodium delivery to the macula densa and thereby decrease renal hyperfiltration. Thus, correction of this early renal hemodynamic dysfunction has the potential to modify the natural history of the disease by curbing the progression of nephropathy. This pathway could not, until now, be adequately studied due to a lack of pharmacologic SGLT-2 inhibitors.

Subjects with T1DM also often face, on top of their chronic hyperglycaemia, excessive glycaemic excursions (despite the use of insulin pump or optimized MDI regimens). SGLT-2 inhibition with BI 10773 is expected to improve glycaemic management in T1DM subjects by 1) improving overall hyperglycaemia (i.e., HbA_{1c}, fasting plasma glucose, postprandial glucose levels and overall glucose exposure) and 2) reducing insulin requirements (due to a lesser amount of circulating glucose, a reduction in body weight and the potential for improved insulin sensitivity). Based on 1) and 2), BI 10773 is also expected to reduce the rate of hypoglycaemia, the risk for hypoglycaemia and glucose excursions. BI 10773 is further expected to reduce or ameliorate weight gain associated with intensification of insulin therapy.

2.2 TRIAL OBJECTIVES

The primary objective of this trial is to determine the impact of 8 weeks of treatment with BI 10773 (25 mg QD) on renal hyperfiltration in subjects with T1DM under conditions of controlled euglycaemia and hyperglycaemia.

The secondary objective of this trial is to characterize the safety and efficacy of BI 10773 (25 mg QD) in subjects with T1DM on insulin pump or multiple daily injections (MDI) therapy.

2.3 BENEFIT - RISK ASSESSMENT

The overall safety profile outlined in Section 1.2 is favourable for human studies at chronic doses up to 50 mg/day. The maximum dose administered in the single rising dose study was 800 mg and the maximum dose for a 4 week treatment was 100 mg/day and both regimes

Proprietary confidential information.
© 2012 Boehringer Ingelheim International GmbH or one or more of its affiliated companies. All rights reserved.
This document may not - in full or in part - be passed on, reproduced, published or otherwise used without prior written permission.

were well tolerated. In Phase II, 12-week treatment duration Trials 1245.9 and 1245.10 in T2DM subjects, the maximum daily dose of BI 10773 was 50 mg. All doses of BI 10773 were well tolerated in these studies.

Because of the insulin-independent mechanism of action of BI 10773 and in light of the fact that GFR is physiologically decreased during episodes of hypoglycaemia (due to reduced renal blood flow hence leading to lowering of UGE and therefore efficacy of BI 10773), the risk of hypoglycaemic episodes with BI 10773 alone is considered to be low. A daily dose of 25 mg BI 10773 has been previously shown to result in urinary excretion of nearly 80 g of glucose every 24 hours. Hence, to adjust the daily insulin requirements in anticipation of the observed increased glucosuria, study subjects will be required to reduce their bolus insulin injections based on Table 2.3: 1 (below); this measure further limits the potential for hypoglycaemia to occur. The estimates for the anticipated required reduction in the amount of bolus insulin doses at the start of treatment (Day 3) are based on the 500 Rule (for Humalog or NovoLog) or the 450 rule (for regular insulin). As an added safety measure, subjects will also be required to decrease their insulin basal dose by 30% at the start of treatment in order to potentially prevent the possible risk of nocturnal hypoglycaemia (based on Table 2.3: 2). If subjects are on a different basal to bolus breakdown than the below examples, the correct reduction amount of basal and bolus insulin will be determined by the investigator.

Table 2.3: 1 Percent reduction in the amount of each daily bolus insulin injection to account for the daily urinary excretion of 80 grams of sugar

Total Daily Insulin Dose (TDD) In Units	The 500 Rule: Grams of Carbs Covered by **1 Unit of Humalog**	Units of insulin needed to cover 80 grams of sugar	**% reduction in amount of daily bolus insulin to account for 80g urinary sugar excretion per below basal to bolus breakdowns**		
			40% Basal **60% Bolus**	45% Basal **55% Bolus**	50% Basal **50% Bolus**
20	25	3.2	**27%**	**29%**	**32%**
25	20	4.0	**27%**	**29%**	**32%**
30	17	4.7	**26%**	**29%**	**31%**
35	14	5.7	**27%**	**30%**	**33%**
40	13	6.2	**26%**	**28%**	**31%**
50	10	8.0	**27%**	**29%**	**32%**
60	8	10.0	**28%**	**30%**	**33%**
**Average bolus reduction:**			**27%**	**29%**	**32%**

The 450 Rule: Grams of Carb Covered by **1 Unit of Regular**	Units of insulin needed to cover 80 grams of sugar	**% reduction in amount of daily bolus insulin to account for 80g urinary sugar excretion per below basal to bolus breakdowns**		
		40% Basal **60% Bolus**	45% Basal **55% Bolus**	50% Basal **50% Bolus**
23	4.0	**29%**	**32%**	**35%**
18	3.2	**30%**	**32%**	**36%**
15	2.7	**30%**	**32%**	**36%**
13	2.3	**29%**	**32%**	**35%**
11	2.0	**30%**	**33%**	**36%**
9	1.6	**30%**	**32%**	**36%**
8	1.3	**28%**	**30%**	**33%**
		**29%**	**32%**	**35%**

Proprietary confidential information.
© 2012 Boehringer Ingelheim International GmbH or one or more of its affiliated companies. All rights reserved.
This document may not - in full or in part - be passed on, reproduced, published or otherwise used without prior written permission.

Table 2.3: 2 The adjusted daily basal insulin amount (in units) for added safety based on a 30% reduction and per below basal to bolus breakdowns

Total Daily Insulin Dose (TDD) In Units	Adjusted daily basal insulin amount (units) for added safety based on a 30% reduction and per below basal to bolus breakdowns		
	40% Basal 60% Bolus	45% Basal 55% Bolus	50% Basal 50% Bolus
20	5.6	6.3	7.0
25	7.0	7.9	8.8
30	8.4	9.5	10.5
35	9.8	11.0	12.3
40	11.2	12.6	14.0
50	14.0	15.8	17.5
60	16.8	18.9	21.0

As CGM will be used in all subjects, a hypoglycaemia threshold alarm will be programmed in all CGM devices (for example 2.2 mmol/l) to prompt subjects to intervene by adjusting their insulin level in case needed or by eating carbohydrates should their glucose level fall to unsafe levels. *All treatment decisions must always be based on confirmed blood glucose levels as determined by fingerstick readings using a home blood glucose meter.*

During the study, all subjects must follow an appropriate investigator-recommended carbohydrate counting method in conjunction with an insulin titration algorithm during the course of the study and per local guidelines in order to achieve optimal and individualized glycaemic goals. Symptoms attributed to hypoglycaemia as well as glucose levels will be closely monitored and appropriate measures will be taken to correct or prevent such occurrences. In addition, based on results from phase II studies in subjects with T2DM, beneficial effects including an improvement in HbA_{1c}, reduced glucose excursions, better overall glucose control, moderate reduction in weight and BP are expected with use of BI 10773.

As with all drugs, the potential for hypersensitivity and allergic reactions has to be taken into consideration when BI 10773 is administered. Other risks to the subjects are the risks inherent to any early clinical trial such as unexpected adverse clinical or laboratory events.

In the embryo-foetal and fertility studies in rats and rabbits, no effects on early embryonic development, mating, male and female fertility, and bearing live young were observed up to a dose of 300 mg/kg. Therefore, women who are of child-bearing potential will be included in this study provided that they are using adequate contraceptive methods.

All subjects participating in this trial will be treated in accordance to the best standard of care in compliance with local guidelines and recommendations. Subjects may derive general medical benefit from careful and close monitoring by medical personnel during the study (including the opportunity to use a continuous glucose monitoring system). Subjects may also derive a direct benefit from being treated with an active compound on top of their standard insulin treatment. The subjects will receive the investigational medication BI 10773 that has already demonstrated favorable HbA_{1c} and glucose changes at the 25 mg dose. Safety will be

Proprietary confidential information.
© 2012 Boehringer Ingelheim International GmbH or one or more of its affiliated companies. All rights reserved.
This document may not - in full or in part - be passed on, reproduced, published or otherwise used without prior written permission.

carefully assessed by monitoring the subjects for adverse events (AEs) clinically, by laboratory testing and by the continuous and home blood glucose monitoring systems. Special measurements will be performed, such as follow-up on genito-urinary tract infections (urine culture), in order to evaluate if possible side effects observed for other SGLT-2 inhibitors are also present for BI 10773. The investigator will have the discretion to remove subjects from the study should there be any safety concerns or if the subject's wellbeing is at jeopardy (example, in cases of repeated episodes of hypoglycaemia).

The potency, selectivity, and efficacy in various animal models and the pharmacodynamic data from studies in healthy volunteers and subjects with diabetes suggest that BI 10773 may be able to provide a valuable benefit to subjects with T1DM. Given the good safety profile in the toxicity studies of BI 10773 and the good tolerability seen in the human studies with up to 84 days treatment and doses up to 100 mg/day, the careful monitoring of subjects during the study, the blood glucose monitoring performed by the subjects at home and the absence of any restriction on antidiabetic therapy in addition to the ability to adjust insulin requirements based on need, the sponsor feels the risks to the participants are minimal and justified when compared with the potential benefits that a successful clinical development of BI 10773 could provide for subjects with T1DM.

Proprietary confidential information.
© 2012 Boehringer Ingelheim International GmbH or one or more of its affiliated companies. All rights reserved.
This document may not - in full or in part - be passed on, reproduced, published or otherwise used without prior written permission.

3. DESCRIPTION OF DESIGN AND TRIAL POPULATION

3.1 OVERALL TRIAL DESIGN AND PLAN

This open-label, single-centre pilot study aims to evaluate the impact of BI 10773 treatment on glomerular filtration rate under controlled conditions of euglycaemia and hyperglycaemia in subjects with T1DM with or without renal hyperfiltration and to characterize the safety and efficacy of BI 10773 25 mg QD as add-on therapy to insulin in these subjects.

In total, approximately 30 subjects with T1DM who meet the entry criteria are planned for inclusion in this trial. Subjects are included in the study once they have signed the informed consent. All subjects suitable after screening will undergo a two week open-label placebo run-in period before the start of the treatment phase. Subjects who successfully complete the run-in period and who still meet the inclusion/exclusion criteria will be entered in the study and will receive BI 10773 25 mg QD for 8 weeks. Before the start of the daily treatment phase and following the successful completion of the run-in period, subjects will be admitted to the renal physiology laboratory at the Toronto General Hospital (study Days 1 and 2), where baseline parameters of their renal and systemic vascular functions will be assessed under controlled conditions of euglycaemia (Day 1) and hyperglycaemia (Day 2). Subjects will be instructed to start taking 25 mg BI 10773 in the morning of Day 3. Subjects will then continue to receive daily administration of 25 mg BI 10773 for approximately 8 weeks while attending clinic and telephone visits as outlined in the trial Flow Chart. Subjects will return to the renal physiology laboratory for another 2 days of renal assessments (study Days 57 and 58) where renal hemodynamic functions and systemic vascular parameters will be measured after administration of the trial drug at the clinic as detailed in the study Flow Chart.

Subject participation is concluded when a subjects has completed the last planned study visit.

The time period, for which AEs will still be considered on-treatment is ≤ 7 days following last intake of trial medication. After trial completion, unresolved AEs are followed-up for up to 30 days and it should be confirmed if they have resolved or have been sufficiently characterised.

The end of the trial is defined as "last subject out", i.e. last visit completed by the last subject (Visit 15).

3.1.1 Administrative structure of the trial

This trial is sponsored by Boehringer Ingelheim (Canada) Ltd/Ltée.

Boehringer Ingelheim (BI) will appoint a Trial Clinical Monitor (TCM), responsible for coordinating the activities required in order to manage the trial in accordance with applicable regulations and internal standard operating procedures (SOPs), directing the clinical trial team in the preparation, conduct, and reporting of the trial, ordering the materials as needed for the trial, ensuring appropriate training and information of a local clinical monitor (CML), clinical research associates (CRAs), and investigators.

Proprietary confidential information.
© 2012 Boehringer Ingelheim International GmbH or one or more of its affiliated companies. All rights reserved.
This document may not - in full or in part - be passed on, reproduced, published or otherwise used without prior written permission.

Data Management and Statistical evaluation will be performed by BI according to BI SOPs. For these activities, a Trial Data Manager and a Trial Statistician will be appointed.

Tasks and functions assigned in order to organise, manage, and evaluate the trial will be defined according to BI SOPs. A list of responsible persons will be given in the Clinical Trial Master File (CTMF) document.

The organisation of the trial will be done by the respective local BI-organisation (Operating Unit (OPU)) or a by a Contract Research Organisation (CRO) with which the responsibilities and tasks will have been agreed and a written contract filed before initiation of the clinical trial. In Canada, a CML will be appointed and will be responsible for coordinating the activities required in order to manage the trial in accordance with applicable regulations and internal SOPs.

Documents on the participating (Principal) investigator and other important participants, especially their curricula vitae, will be filed in the CTMF.

Details on handling of the trial supplies including responsible institutions are given in Section 4 of this protocol.

The Investigator Site File (ISF) will be kept at the site as required by local regulation and BI-SOP. A copy of the ISF documents will be kept as an electronic CTMF document according to BI SOPs.

Project DMC:

A project based data-monitoring committee (DMC), independent from the sponsor will be established to assess the progress of all core Phase III clinical trials, including the safety data and the critical efficacy endpoints at intervals, and to recommend to the sponsor whether to continue, modify, or stop one or all trials involved. Data from this pilot study will also be forwarded to the DMC.

The tasks and responsibilities of the DMC will be filed in a contract before initiation of the trial and will contain written operating procedures. The DMC will maintain written records of all its meetings.

3.2 DISCUSSION OF TRIAL DESIGN, INCLUDING THE CHOICE OF CONTROL GROUP

This trial is a single centre mechanistic study where eligible subjects, diagnosed with T1DM, on insulin pump therapy or multiple daily injections (MDI), will receive open-label BI 10773 (25 mg QD) for approximately 8 weeks of treatment. The scope of the design of this trial is to assess the impact of BI 10773 administration on renal function under controlled laboratory conditions of euglycaemia and hyperglycaemia, as well as to characterize the safety and efficacy of BI 10773 when administered on top of CSII or MDI treatment in subjects with T1DM. For the purpose of the renal assessments and analyses, subjects will be stratified in 2 groups based on their baseline GFR measured under controlled euglycaemia at Visit 3 as

Proprietary confidential information.
© 2012 Boehringer Ingelheim International GmbH or one or more of its affiliated companies. All rights reserved.
This document may not - in full or in part - be passed on, reproduced, published or otherwise used without prior written permission.

assessed by inulin measurements (non-hyperfilterers will be subjects with a measured GFR of ≥ 60 ml/min/1.73m² to < 135 ml/min/1.73m² and hyperfilterers will be subjects with a measured GFR ≥ 135 ml/min/1.73m²). Due to its mechanism of action, BI 10773 is expected to provide additional benefits in combination to background insulin and to reduce a subject's renal hyperfiltration state by correcting early renal hemodynamic dysfunctions.

All subjects will receive the trial medication in an open-label fashion and on top of their current daily insulin requirements.

The estimation of the sample size is based on the expected change in GFR in hyperfilterers and non-hyperfilterers in similar previously conducted studies. As the results of the GFR will not be available until 3-4 weeks after the completion of the renal assessments during Visit 3, recruitment may continue until a minimum of 12 hyperfilterer subjects and 12 non-hyperfilterer subjects have completed the study (Visit 13). The rational for this minimum is based on the sample size calculations. An interim analysis is not planned in this study. The treatment period is 8 weeks. A 2-week follow-up period is considered to be sufficient, as previous studies with BI 10773 have shown that the pharmacodynamic effect of BI 10773 only extends to about three days after the last administered dose.

The rationale for dose and dose-interval selection is described in Section 4.1.3.

3.3 SELECTION OF TRIAL POPULATION

It is estimated that approximately 45 subjects will be screened at a single centre in Canada, and it is expected that 30 subjects will be entered in the trial and will receive the trial drug. All subjects will receive placebo run-in tablets during the 2 weeks preceding the 8-week treatment period where all entered subjects will receive open-label BI 10773 25 mg QD.

Screening of subjects may be stopped when at least 12 subjects in the hyperfilterer group and 12 subjects in the non-hyperfilterer group have completed the study. Subjects will be stratified in these 2 groups based on their baseline GFR, measured under controlled euglycaemia during Visit 3 (non-hyperfilterers will have GFRs of ≥ 60 ml/min/1.73m² to < 135 ml/min/1.73m² and hyperfilterers will have GFRs of ≥ 135 ml/min/1.73m²). Subject eligibility will be assessed based upon a complete medical history including a physical examination and clinical laboratory tests. Judgement of the clinical relevance of a concomitant disease is at the discretion of the investigator. Conditions under therapy are always clinically relevant.

A log of all subjects included into the study (i.e. having given informed consent) will be maintained in the ISF at the investigational site irrespective of whether they have been treated with the investigational drug or not.

3.3.1 Main diagnosis for study entry

This mechanistic pilot study will be performed in adult subjects with T1DM who are on insulin pump therapy or MDI treatment and have an eGFR ≥ 60 ml/min/1.73m² at screening.

Proprietary confidential information.
© 2012 Boehringer Ingelheim International GmbH or one or more of its affiliated companies. All rights reserved.
This document may not - in full or in part - be passed on, reproduced, published or otherwise used without prior written permission.

3.3.2 Inclusion criteria

1. Male or female subject diagnosed with T1DM at least 12 months prior to informed consent
2. Age ≥ 18 years
3. Signed and dated written informed consent by date of Visit 1 in accordance with GCP and local legislation
4. HbA_{1C} of 6.5% to 11.0% at Visit 1 (no more than 5 subjects with an HbA_{1C} between 6.5% to <7.0% will be included)
5. BMI (Body Mass Index) of 18.5 kg/m^2 to 35.0 kg/m^2 at Visit 1 (Screening)
6. Subjects must be either:
    - experienced insulin pump users (≥ 3 months of use prior to Visit 3 and willing to use the same insulin pump during the course of the study) or
    - be on multiple daily injections (MDI) of any type of insulin (and be willing to continue on MDI therapy during the course of the study)
7. Subjects must follow an established and individualized carbohydrate counting method and an insulin titration algorithm based on investigator recommendations
8. Subjects must have had a stable glycaemic status defined by their latest HbA_{1C} value as measured 2-12 months prior to screening which must be within 1.5% of the baseline screening value (Visit 1)
9. Subjects must have an eGFR ≥ 60 ml/min/1.73m^2 at screening
10. Subject must be able and willing to perform study assessments

3.3.3 Exclusion criteria

1. Evidence of macroalbuminuria (spot urine albumin to creatinine ratio > 300 mg/g of creatinine)
2. Leukocyte and/or nitrite positive urinalysis at screening (Visit 1). Subjects may be re-screened if the condition is treated
3. Any concomitant medication known to interfere with RAAS activity and/or renal function based on investigator judgement. Examples of restricted drug classes include angiotensin-converting enzyme (ACE) inhibitors, angiotensin receptor blockers (ARBs), aldosterone antagonists and renin inhibitors
4. T1DM treatment with any other drugs to reduce blood glucose except insulin within 6 months prior to screening (example: off-label use of pramlintide or metformin)

Proprietary confidential information.
© 2012 Boehringer Ingelheim International GmbH or one or more of its affiliated companies. All rights reserved.
This document may not - in full or in part - be passed on, reproduced, published or otherwise used without prior written permission.

5. Occurrence of severe hypoglycaemia that required emergency hospital treatment over the past 3 months prior to Visit 1

6. History of macrovascular disease inclusive of cardiovascular, cerebrovascular and peripheral artery disease

7. Known autonomic neuropathy and proliferative retinopathy including treated proliferative retinopathy. Subjects with mild nonproliferative diabetic retinopathy can be included

8. Diagnosis of brittle diabetes based on investigator judgement

9. Hypoglycaemia unawareness based on investigator judgement

10. Total daily insulin requirement > 1.5 U/kg at screening

11. Pancreas, pancreatic islet cells or renal transplant recipient

12. Indication of liver disease, defined by serum levels of either alanine transaminase (ALT) (SGPT), aspartate transaminase (AST) (SGOT), or alkaline phosphatase above 3 x upper limit of normal (ULN) as determined during screening and/or the run-in phase

13. Bariatric surgery within the past two years and other gastrointestinal surgeries that induce chronic malabsorption

14. Treatment with anti-obesity drugs (e.g. sibutramine, orlistat), surgery or aggressive diet regimen leading to unstable body weight three months prior screening (Visit 1)

15. Treatment with systemic steroids at the time of informed consent

16. Medical history of cancer or treatment for cancer in the last five years prior to the Visit 1. Fully treated basal cell carcinoma is exempted

17. Blood dyscrasias or any disorders causing hemolysis or unstable red blood cells (e.g. malaria, babesiosis, hemolytic anemia)

18. Pre-menopausal women (last menstruation ≤ 1 year prior to informed consent) who:

    a. are nursing or pregnant or

    b. are of child-bearing potential and are not practising an acceptable method of birth control, or do not plan to continue using this method throughout the study and do not agree to submit to periodic pregnancy testing during participation in the trial. Acceptable methods of birth control include tubal ligation, transdermal patch, intra uterine devices/systems (IUDs/IUSs), oral, implantable or injectable contraceptives, sexual abstinence, double barrier method and vasectomised partner

Proprietary confidential information.
© 2012 Boehringer Ingelheim International GmbH or one or more of its affiliated companies. All rights reserved.
This document may not - in full or in part - be passed on, reproduced, published or otherwise used without prior written permission.

19. Participation in another trial with an investigational drug within 30 days prior to informed consent

20. Alcohol or drug abuse within the three months prior to informed consent that would interfere with trial participation based on investigator judgement or any ongoing clinical condition that would jeopardize subject safety or study compliance based on investigator judgement

3.3.4 Removal of subjects from therapy or assessments

3.3.4.1 Removal of individual subjects

An individual subject is to be withdrawn from the trial if:

- The subject withdraws consent, without the need to justify the decision
- The subject needs to take concomitant drugs that interfere with the investigational product or planned study assessments
- The subject is no longer able to participate for other medical reasons (e.g. surgery, AEs or other diseases)
- Occurrence of hypoglycaemia that may put the subject at risk with continued participation (e.g. repeated hypoglycaemic episodes) based on investigator judgement
- If a subject becomes pregnant during the trial the investigational drug will be stopped, the subject will be discontinued from the trial and the subject will be followed up until birth or otherwise termination of the pregnancy.

A subject can be discontinued after discussion between sponsor and investigator if eligibility criteria are being violated, or if the subject fails to comply with the protocol (e.g. non-attendance at study assessments).

Subjects who drop out during the screening phase and before the start of treatment with BI 10773 (study Day 3) will be considered as screening failures. They have to be recorded as a screening failure in the electronic Case Report Forms (eCRFs) and no further follow-up is required.

Subjects who discontinue or withdraw from the study after study Day 3 (after the start of therapy) will be considered as "early discontinuations" and the reason for premature discontinuation will be recorded in the eCRFs. Subjects who discontinue prematurely (before completion of Visit 13) will be replaced in order to obtain at least 12 subjects in the hyperfilterer group and 12 in the non-hyperfilterer group who complete the treatment phase of the study (Visit 13). The data for screen failures will be included in the trial database and will be reported. A new antidiabetic medication regimen can be started immediately after discontinuation, if determined by the investigator as necessary for subject safety. This must be recorded in the eCRFs.

Proprietary confidential information.
© 2012 Boehringer Ingelheim International GmbH or one or more of its affiliated companies. All rights reserved.
This document may not - in full or in part - be passed on, reproduced, published or otherwise used without prior written permission.

For early discontinued subjects, the required assessments under the EOT visit should be performed. Refer to the study Flow Chart (under the EOT visit) or Section 6.2.3 for details. Early discontinued subjects should return to the clinic for a follow up visit (only Visit 15 is required).

3.3.4.2 Discontinuation of the trial by the sponsor

Boehringer Ingelheim reserves the right to discontinue the trial overall at any time for the following reasons:

1. Failure to meet expected enrolment goals overall,
2. Emergence of any efficacy/safety information that could significantly affect continuation of the trial and/or invalidate the earlier positive benefit-risk-assessment
3. Violation of GCP, the CTP, or the contract the trial site or investigator, disturbing the appropriate conduct of the trial.

The investigator / the trial site will be reimbursed for reasonable expenses incurred in case of trial termination (except in case of the third reason).

Proprietary confidential information.
© 2012 Boehringer Ingelheim International GmbH or one or more of its affiliated companies. All rights reserved.
This document may not - in full or in part - be passed on, reproduced, published or otherwise used without prior written permission.

4. TREATMENTS

4.1 TREATMENTS TO BE ADMINISTERED

The study medication will be provided by Boehringer Ingelheim.

4.1.1 Identity of BI investigational product and comparator product

The characteristics of the products are listed below (BI 10773 will only be administered during the open-label treatment period and placebo matching BI 10773 25 mg will only be administered during the 2 week run-in period).

Substance:	BI 10773
Pharmaceutical form:	tablet
Source:	Boehringer Ingelheim
Unit Strength:	25 mg
Route of administration:	p.o., once daily

Substance:	placebo matching BI 10773 25 mg
Pharmaceutical form:	tablet
Source:	Boehringer Ingelheim
Unit Strength:	-
Route of administration:	p.o., once daily

There are no comparator products in this study.

4.1.2 Method of assigning subjects to treatment groups

Once the criteria for entry into the treatment period of the study are confirmed, BI 10773 at a dose of 25 mg once daily will be given to subjects in an open-label fashion. The investigative site will receive separate treatment kits for the run-in period and the treatment phase.

Proprietary confidential information.
© 2012 Boehringer Ingelheim International GmbH or one or more of its affiliated companies. All rights reserved.
This document may not - in full or in part - be passed on, reproduced, published or otherwise used without prior written permission.

4.1.3 Selection of doses in the trial

BI 10773 will be administered in a 25 mg dose once daily. This dose was selected based on results from previous dose finding studies (refer to the current version of the Investigator's Brochure for additional information).

4.1.4 Drug assignment and administration of doses for each subject

The treatment to be evaluated is outlined in Table 4.1.4: 1. Subjects who qualify will receive the run-in medication and treatment medication as indicated below. Medication will be dispensed in an open-label fashion.

All subjects will be assigned an open-label placebo run-in medication kit just once at the beginning of the placebo run-in period (Visit 2). Dispensing of kits for the open-label treatment period will be done at the end of Visit 4 and subject will be instructed to take their first dose of study medication in the morning of study Day 3. Dispensing of BI 10773 will occur on 2 occasions over a period of 8 weeks. For further details regarding packaging (e.g. number of tablets per container) refer to Section 4.1.6.

Table 4.1.4: 1 Timing and dosage of BI 10773 25 mg and matching placebo

	**All subjects**	**Total units per dose**	**Timing**
**Placebo run-in period**	matching placebo 25 mg BI 10773	1 tablet	once daily, morning (7:00 AM – 11:00 AM)
**Treatment period (open-label)**	25 mg BI 10773	1 tablet	once daily, morning (7:00 AM – 11:00 AM)

From the start of the placebo run-in period (Visit 2), subjects should be instructed to take their trial medication once daily with water in the morning between 7:00 AM – 11:00 AM, except during study days 57 and 58 when the trial medication will be given at a later time as specified in the study Flow Chart (around 12:00 PM). To ensure a dose interval of approximately 24 hours, the medication should be taken at the same time every day. If a dose is missed by more than 12 hours, that dose should be skipped and the next dose should be taken as scheduled. On the day prior to a visit, the dose should be taken 22-26 h before the planned dose at the visit (except for Visit 12 and Visit 13 when subject will be dose at the clinic 1.5 hours before the start of renal assessments). No double doses should be taken and dose reductions are not permitted. BI 10773 can be taken with or without food. The first dose of BI 10773 will be taken in the morning of study Day 3 after baseline renal assessments have been completed (after Visit 3 and Visit 4). Thereafter, subjects will be instructed to take

Proprietary confidential information.
© 2012 Boehringer Ingelheim International GmbH or one or more of its affiliated companies. All rights reserved.
This document may not - in full or in part - be passed on, reproduced, published or otherwise used without prior written permission.

one tablet of BI 10773 25 mg daily in the morning. The last dose of BI 10773 will be administered 1.5 hours prior to renal assessments at Visit 13.

Subjects should be instructed not to take their trial medication on the morning of study clinic visits as they will be dosed at the clinic if required. Visits should be routinely scheduled in the morning, at approximately the same time of day (07:00 AM to 11:00 AM) for each visit (except Visits 3, 4, 12 and 13). The actual date and time of administration of the medication at the trial visit will be recorded in the eCRF.

The investigator or delegate may choose any available kit number for dispensing. A run-in medication kit should only be dispensed during the run-in phase and a treatment kit should only be dispensed to an eligible subject once the subject has completed the run-in phase and is still eligible for the study. The visit boxes from the same treatment medication kit should be dispensed to a subject at Visit 4 and Visit 8. All medication kit numbers will be recorded in eCRFs.

4.1.5 Blinding and procedures for unblinding

4.1.5.1 Blinding

This is an open-label study.

4.1.5.2 Procedures for emergency unblinding

This is an open-label study.

4.1.6 Packaging, labelling, and re-supply

Drug supplies will be provided by the Department of Pharmaceutical Development of Boehringer Ingelheim Pharma GmbH & Co. KG, Biberach, Germany. All study medication will be contained in medication boxes identified with the trial number and medication number. Each medication box will contain an appropriate amount of BI 10773 or placebo tablets, plus some reserve, for dosing until the next scheduled visit.

Each run-in kit will contain three child-resistant wallet cards. Each placebo wallet card will contain 7 tablets with placebo to match BI 10773 25 mg. Each treatment kit will contain 2 visit boxes (dispensed at Visits 4 and 8). Each visit box will contain six child-resistant wallet cards. Each treatment wallet card will contain 7 tablets of BI 10773 25 mg.

Enough trial medication will be supplied to the subject to last until the planned next clinic visit.

4.1.7 Storage conditions

The trial medication (BI 10773 and placebo) must be kept in its closed original packaging under the recommended storage conditions indicated on the label. The minimum/maximum storage temperature must be measured and documented by the Investigator / pharmacist / investigational drug storage manager in accordance with BI SOPs. If storage conditions are

Proprietary confidential information.
© 2012 Boehringer Ingelheim International GmbH or one or more of its affiliated companies. All rights reserved.
This document may not - in full or in part - be passed on, reproduced, published or otherwise used without prior written permission.

found to be outside the specified range, site personnel should immediately contact the CML via the list of contacts in the ISF.

4.1.8 Drug accountability

Trial medication, which will be provided by the Sponsor and must be kept in a secure, limited access storage area under the storage conditions defined by the Sponsor. A temperature log must be maintained to make certain that the drug supplies are stored at the correct temperature.

The Investigator / pharmacist / investigational drug storage manager will receive the investigational drugs delivered by the Sponsor when the following requirements are fulfilled:

- approval of the study protocol by the Institutional Review Board (IRB) / Independent Ethics Committee (IEC),
- availability / completion of a signed and dated clinical trial contract between the Sponsor and the Head of the Trial Centre or a representative from the Trial Centre as appropriate,
- approval / notification of the regulatory authority, e.g. competent authority (CA),
- availability of the curriculum vitae of the Principal Investigator,
- availability of a signed and dated clinical trial protocol (CTP) or immediately imminent signing of the CTP
- availability of the proof of a medical licence for the Principal Investigator

The Investigator / pharmacist / investigational drug storage manager must maintain records of the delivery of the trial medication to the trial site, the inventory at the site, the use by each subject, and the return to the Sponsor or alternative disposition of unused medication.

These records will include dates, quantities, batch/serial numbers, expiry ('use by') dates, and the unique code numbers assigned to the investigational products and trial subjects. The Investigator / pharmacist / investigational drug storage manager will maintain records that document adequately that the subjects were provided the doses specified by the CTP and reconcile all investigational product received from the Sponsor. At the time of return to the Sponsor / appointed CRO, the Investigator / pharmacist / investigational drug storage manager must verify that all unused or partially used drug supplies have been returned by the clinical trial subject and that no remaining supplies are in the Investigator's possession.

4.2 CONCOMITANT THERAPY, RESTRICTIONS, AND RESCUE TREATMENT

Details of all concomitant therapy during the clinical trial will be recorded on the appropriate pages of the eCRFs.

Proprietary confidential information.
© 2012 Boehringer Ingelheim International GmbH or one or more of its affiliated companies. All rights reserved.
This document may not - in full or in part - be passed on, reproduced, published or otherwise used without prior written permission.

4.2.1 Rescue medication, emergency procedures and additional treatments

During the screening and run-in period, subjects should continue to take the antidiabetic treatment they were receiving at informed consent as background therapy. The background insulin medication can remain unchanged or change partially or completely as per subject needs or based on the investigator's clinical judgement (treatment to target according to local guidelines is encouraged).

Insulin will not be provided as part of the clinical trial supplies.

To prevent the possible risk of hypoglycaemic episodes in subjects with T1DM on insulin therapy and BI10773, study subjects will initially be required to reduce their basal and bolus insulin injections by approximately 30% at the start of the treatment phase (one day after Visit 4 – study Day 3). All subjects must follow an appropriate investigator-recommended carbohydrate counting method and insulin titration algorithm during the course of the study in order to achieve optimal and individualized glycaemic goals. It is expected that insulin requirements will change during the course of the treatment phase and as such, each subject will have to titrate their insulin intake accordingly to prevent episodes of hyper or hypoglycaemia. Insulin dose adjustments should be done when feasible under the investigator's guidance. In addition, subject will have to test their blood glucose level at home several times a day as recommended by the investigator. *Any insulin dose change or adjustment must be based on blood glucose measurements (not CGM data).*

In case of hypoglycaemia, which may put a subject at risk (for example, repeated symptomatic hypoglycaemia or severe hypoglycaemia), appropriate reduction of ongoing background therapy (insulin) should be initiated in consultation with the investigator when feasible.

There are no special emergency procedures to be followed. There are no rescue medications in this trial. The patient's background insulin dose can change at any time in order to achieve the best standard of care.

4.2.2 Restrictions

4.2.2.1 Restrictions regarding concomitant treatment

Any treatment that is known to interfere with RAAS activity and/or renal function is strictly prohibited. Such medications include angiotensin-converting enzyme (ACE) inhibitors, angiotensin receptor blockers (ARBs), aldosterone antagonists and renin inhibitors or any other medication / drug class per investigator judgement which may interfere with RAAS activity and/or renal function.

Treatment with anti-obesity drugs or systemic steroids will be prohibited due to their influence on glucose metabolism. However, short-term use (i.e. ≤ 3 days duration) of systemic steroids, if deemed appropriate by the investigator, will be permitted as well as therapy with non-systemic steroids such as inhaled or local steroids. For subjects taking

Proprietary confidential information.
© 2012 Boehringer Ingelheim International GmbH or one or more of its affiliated companies. All rights reserved.
This document may not - in full or in part - be passed on, reproduced, published or otherwise used without prior written permission.

thyroid hormones, any change in the dose should be avoided. If dose changes do occur, then they should be recorded in the source documents and in the eCRF.

4.2.2.2 Restrictions on diet and life style

At the beginning of the Run-in period (i.e. at Visit 2), subjects will receive diet and exercise counselling by the Investigator or a member of the clinic staff. The counselling will be based on local diet recommendations for subjects with T1DM in consideration with each subject's possible concomitant illnesses. Site specific tools may be used to help subjects with diet and life style / exercise counselling. The subjects will be reminded to follow the recommended diet and exercise plan during the study. Subject are also requested to adhere to a specific high-sodium (preferably > 140 mmol/day) and moderate-protein (< 1.5 g/kg/day) diet only during the 7 days preceding the start of renal physiology laboratory assessments (respectively 7 days before study Days 1-2 and 57-58). Details of this specific diet will be given to subjects at Visit 2 and subjects will be reminded to re-start this diet at Visit 11.

Women of child-bearing potential must continue to practice an acceptable method of birth control (in accordance with the trial exclusion criteria Section 3.3.3) throughout the duration of the study including the follow up period.

Subjects should also not take part in another clinical trial involving an investigational medicinal product within the last 30 days before the date of informed consent for this trial.

4.3 TREATMENT COMPLIANCE

Subjects will be asked to bring all trial medication containers (with or without any remaining tablets) with them to each trial visit. The tablets will be counted by the Investigator or designee and compliance will be calculated according to the formula:

$$\text{Compliance}(\%) = \frac{\text{Number of tablets actually taken since last tablet count}}{\text{Number of tablets which should have been taken in the same period}} \times 100\%$$

Compliance during the placebo run-in period should be between 80% and 120%. If compliance is outside this range, the subject should be carefully interviewed and, if necessary, re-informed about the purpose and the conduct of the trial. Unreliable subjects should not be entered in the study at the discretion of the Investigator.

Compliance during the treatment period should also be between 80% and 120%. Subjects who are not compliant according to this definition should be carefully interviewed and re-informed about the purpose and the conduct of the trial.

Proprietary confidential information.
© 2012 Boehringer Ingelheim International GmbH or one or more of its affiliated companies. All rights reserved.
This document may not - in full or in part - be passed on, reproduced, published or otherwise used without prior written permission.

5. VARIABLES AND THEIR ASSESSMENT

5.1 EFFICACY - PHARMACODYNAMICS

5.1.1 Endpoints of efficacy

Primary renal endpoint:

The primary exploratory renal endpoint is the change in glomerular filtration rate (GFR) after treatment with BI 10773 under controlled conditions of euglycaemia and hyperglycaemia

Other exploratory renal endpoints:
The other renal exploratory endpoints after treatment with BI 10773 under controlled conditions of euglycaemia and hyperglycaemia include the following:

- Change in renal hemodynamic function: ERPF, RBF, FF and RVR
- Change in systemic hemodynamic function: MAP and arterial stiffness
- Change in circulating levels of mediators involved in RAAS activation and markers of sympathetic activity
- Change in urinary nitric oxide, prostanoids and albumin excretion

Other exploratory diabetes mellitus endpoints:

The other exploratory diabetes endpoints include:

- Change from baseline in HbA_{1c} after 8 weeks of treatment with BI 10773
- Change from baseline in ~~glycated albumin (GA)~~ **fructosamine (FA)** after 8 weeks of treatment with BI 10773
- Change in fasting plasma glucose from baseline
- Change in glycaemic exposure, variability and stability as derived from Continous Glucose Monitoring (CGM) from baseline
- Change in parameters of glucose variability (as measured by CGM or home blood glucose monitoring [HBGM])
- Change in mean daily glucose (as derived from the 8-p HBGM)
- Change in AUC for glucose (CGM data) from baseline
- Change in basal, bolus and total insulin requirement from baseline
- Change in basal insulin requirement day vs night from baseline
- Change in the numbers of bolus boosts from baseline
- Change in UGE from baseline
- Change in BP from baseline
- Change in the Diabetes Treatment Satisfaction Questionnaire from baseline

Proprietary confidential information.
© 2012 Boehringer Ingelheim International GmbH or one or more of its affiliated companies. All rights reserved.
This document may not - in full or in part - be passed on, reproduced, published or otherwise used without prior written permission.

- Change in HbA_{1c}, hypoglycaemia and insulin requirements from baseline (refer to 7.3.2 for details of responder classification)

5.1.2 Assessment of efficacy

5.1.2.1 Hemodynamic measurements

For all experimental phases, blood glucose is maintained by a modified glucose clamp technique, as previously described (R10-6374). In summary, a 16-gauge peripheral venous cannula is inserted into the left antecubital vein for infusion of glucose and insulin and more distally, a second cannula is inserted for blood sampling. Blood glucose is measured every 10–15 min, and the insulin infusion is adjusted to maintain the desired glycaemic index. Experiments are performed in the same temperature controlled room and in a dark, quiet environment after 10 min of rest in the supine position. Renal and systemic vascular function assessments are performed at baseline and then repeated in an identical fashion after 8 weeks of BI 10773 therapy (25 mg orally/day). Following arterial stiffness assessments (more details in Section 5.1.2.2), a third intravenous line is inserted into the right arm and is connected to a syringe infusion pump for inulin and paraaminohippurate (PAH) administration. After collection of blood for inulin and PAH blank, a priming infusion containing 25% inulin (60 mg/kg) and 20% PAH (8 mg/kg) is administered. Thereafter, inulin and PAH are infused continuously at a rate calculated to maintain their respective plasma concentrations constant at 20 and 1.5 mg/dl. After a 90-min equilibration period, blood is collected for inulin, PAH, and hematocrit. Blood is also collected as applicable every 30 mins for 60 mins for inulin and PAH measurements. Glomerular filtration rate (GFR) and effective renal plasma flow (ERPF) are respectively estimated by steady-state infusion of inulin and PAH (R10-6364).

5.1.2.2 Arterial stiffness and sympathetic nervous system (SNS) measurements

Augmentation index (AIx) for the radial and carotid arteries as well as a derived aortic AIx and carotid, radial and femoral pulse wave velocities (PWV) will be measured for assessment of arterial stiffness when feasible using a SphygmoCor® System, and necessary software or a similar instrument based on previously published methodologies (R11-0072). Such an instrument allows a non-invasive means of quantifying cardiac autonomic activity allowing assessment of sympathetic and parasympathetic autonomic function including variable heart rate which will be measured as part of the SNS assessments. Laboratory measurements of adrenaline and noradrenaline will also be done as part of the SNS assessments. The SphygmoCor® System provides a comprehensive assessment of the key cardiac parameters including central blood pressure, arterial stiffness and autonomic function and calculates both time and frequency domain heart rate variability parameters, including the vagal index. The use of the SphygmoCor® System for assessment of arterial stiffness and endothelial function has been previously validated (R10-6380).

Proprietary confidential information.
© 2012 Boehringer Ingelheim International GmbH or one or more of its affiliated companies. All rights reserved.
This document may not - in full or in part - be passed on, reproduced, published or otherwise used without prior written permission.

5.1.2.3 Blood pressure and pulse rate

Blood pressure and pulse rate measurements will be made using an automated Dinamap sphygmomanometer. Measurements will be taken in the supine position during the renal study Days 1, 2, 57 and 58. An average of three measurements will be recorded and used to calculate renal hemodynamic parameters. In addition, upon arrival on Days 1, 2, 57 and 58, postural vitals will be taken. Blood pressure measurements should be recorded to the nearest 2 mmHg only when measured with a manual sphygmomanometer; when digital devices are used, the value from the device should be rounded to the nearest 1 mmHg.

For routine vital sign assessments (for visits other than Visit 3, 4, 12 and 13), systolic and diastolic BP as well as pulse rate will be measured after 5 minutes of rest in the supine position. The BP measurement should be performed three times and all three measurements will be entered in the eCRF. The second and third BP measurements will be done respectively 2 minutes and 4 minutes after the initial measurement. The pulse rate measurement will be done during the 2nd and 3rd BP measurement after a full minute count.

BP measurements should be performed on the same arm after the initial assessment and, if possible, by the same person. The same method and device must be used throughout the trial for a subject.

The initial reading of BP at screening (Visit 1) should be done in both arms. If the pressures differ by more than 10 mmHg (as in the presence of a subclavian steal syndrome), the arm with the higher pressure (systolic or if needed to decide, diastolic) should be used for subsequent measurements.

5.1.2.4 Fasting plasma glucose

Blood samples for the determination of FPG will be taken after an overnight fast. The samples should be taken before breakfast <u>and</u> before trial drug administration if applicable based on the study Flow Chart. The samples will be measured at a local certified laboratory using validated assays and sample handling and shipment will be done per local requirements.

5.1.2.5 Glycated hemoglobin (HbA_{1c}) and ~~glycated albumin (GA)~~ **Fructosamine (FA)**

Blood samples for the determination of HbA_{1c} and ~~GA~~ **FA** will be taken. Samples can be taken in a non-fasted state. The blood samples should be drawn as required by the study Flow Chart before trial drug administration. The samples will be analysed at a local certified laboratory ~~having a National Glycohemoglobin Standardisation Program (NGSP) Level I certificate~~.

5.2 SAFETY

5.2.1 Endpoints of safety

- Incidence of adverse events

Proprietary confidential information.
© 2012 Boehringer Ingelheim International GmbH or one or more of its affiliated companies. All rights reserved.
This document may not - in full or in part - be passed on, reproduced, published or otherwise used without prior written permission.

- Protocol-specified significant adverse events
- Hypoglycaemic events: Occurrence of serious hypoglycaemia, symptomatic hypoglycaemia (mild or moderate) and time spent in CGM defined hypoglycaemia
- Hypoglycaemic events or time spent in CGM-defined hypoglycaemia day vs. night
- Change in frequency of asymptomatic hypoglycaemia <3.0 mmol/l (by home blood glucose monitoring) from baseline
- Change in weight from baseline
- Change in waist circumference
- Changes from baseline in clinical laboratory values (e.g. electrolytes)
- Vital signs

5.2.2 Assessment of adverse events

5.2.2.1 Definitions of adverse events

Adverse event

An AE is defined as any untoward medical occurrence, including an exacerbation of a pre-existing condition, in a subject in a clinical investigation who received a pharmaceutical product. The event does not necessarily have to have a causal relationship with this treatment.

Serious adverse event

A SAE is defined as any AE which results in death, is immediately life-threatening, results in persistent or significant disability / incapacity, requires or prolongs subject hospitalisation, is a congenital anomaly / birth defect, or is to be deemed serious for any other reason if it is an important medical event when based upon appropriate medical judgement which may jeopardise the subject and may require medical or surgical intervention to prevent one of the other outcomes listed in the above definitions.

Intensity of adverse event

The intensity of the AE should be judged based on the following:

Mild:	Awareness of sign(s) or symptom(s) which is/are easily tolerated
Moderate:	Enough discomfort to cause interference with usual activity
Severe:	Incapacitating or causing inability to work or to perform usual activities

Causal relationship of adverse event

Medical judgement should be used to determine the relationship, considering all relevant factors, including pattern of reaction, temporal relationship, de-challenge or re-challenge, confounding factors such as concomitant medication, concomitant diseases and relevant history. Assessment of causal relationship should be recorded in the case report forms.

Proprietary confidential information.
© 2012 Boehringer Ingelheim International GmbH or one or more of its affiliated companies. All rights reserved.
This document may not - in full or in part - be passed on, reproduced, published or otherwise used without prior written permission.

Yes: There is a reasonable causal relationship between the investigational product administered and the AE.
No: There is no reasonable causal relationship between the investigational product administered and the AE.

If a SAE is reported from a still blinded trial, the causal relationship must be provided by the investigator for all potential trial drugs, i.e. the BI trial drug and for all other trial drugs.

Worsening of underlying disease or other pre-existing conditions

Expected fluctuations or expected deterioration of the underlying disease and other pre-existing conditions should not be recorded as an AE unless at least one of the following criteria is met:

- the worsening of the disease constitutes an SAE
- the investigational drug is discontinued or the dose is reduced or increased
- additional treatment is required, i.e. concomitant medication is added or changed
- An unexpected deterioration from baseline has occurred in the opinion of the investigator.

Changes in vital signs, ECG, physical examination, and laboratory test results

Changes in vital signs including blood pressure, pulse rate, ECG, physical examination, and laboratory tests will be only then recorded as AEs if they are not associated with an already reported AE, symptom or diagnosis, and the investigational drug is either discontinued, reduced or increased, or additional treatment is required, i.e. concomitant medication is added or changed.

Significant Adverse Events

Irrespective of whether an AE is serious or non-serious, the following events are defined as 'protocol-specified significant adverse events':

- Decreased renal function: creatinine value shows a ≥2 fold increase from baseline and is above the upper limit of normal.
- Hepatic injury defined by the following alterations of liver parameters: An elevation of AST and /or ALT above ≥ 3 fold ULN combined with an elevation of bilirubin above ≥ 2 fold ULN measured in the same blood sample. Subjects showing these lab abnormalities need to be followed up appropriately.

In such a case, the event should be reported to the drug safety centre immediately being documented on an SAE form. The investigator shall collect an unscheduled laboratory sample for creatinine or hepatic enzymes as soon as possible and initiate follow-up laboratory test of creatinine or hepatic enzymes at the next visits as appropriate.

Proprietary confidential information.
© 2012 Boehringer Ingelheim International GmbH or one or more of its affiliated companies. All rights reserved.
This document may not - in full or in part - be passed on, reproduced, published or otherwise used without prior written permission.

Expected AEs

The Investigator's Brochure will list the events that regulatory wise are considered expected events for BI 10773. At the time of the CTP finalisation, no expected AEs were identified.

5.2.2.2 Adverse event and serious adverse event reporting

All adverse events, serious and non-serious, occurring during the course of the clinical trial (i.e., from signing the informed consent onwards through the observational phase) will be collected, documented and reported to the sponsor by the investigator on the appropriate CRFs / eCRFs / SAE reporting forms. Reporting will be done according to the specific definitions and instructions detailed in the 'Adverse Event Reporting' section of the Investigator Site File.

For each AE, the investigator will provide the onset date, end date, intensity, treatment required, outcome, seriousness, and action taken with the investigational drug. The investigator will determine the relationship of the investigational drug to all AEs as defined in Section 5.2.2.1.

Clinically relevant abnormalities found during the physical examination at Visit 1 (including abnormalities with vital signs or ECG) will be considered to have already existed prior to signing of informed consent and therefore should be considered baseline conditions instead of adverse events, unless there is good reason to assume that they first appeared after signing of informed consent.

The investigator also has the responsibility to report AEs occurring within one week after a subject completes the trial. Any AEs reported to the sponsor during this period must be documented in the safety database.

If not stipulated differently in the ISF, the investigator must report the following events via telephone/fax using the SAE form immediately (within 24 hours or the next business day whichever is shorter) to the sponsor: SAEs and non-serious AEs occurring at the same time as an SAE and/or which are medically related to the SAE(s), and protocol-specified significant events. With receipt of any further information to these events, a follow-up SAE report has to be provided. SAEs and non-serious AEs must include a causal relationship assessment made by the investigator.

The SAE form is to be forwarded to the defined unique entry point identified for the BI OPU (country-specific contact details will be provided in the ISF). This immediate report is required irrespective of whether the investigational product has been administered or not and irrespective of causal relationship. It also applies if new information to existing SAEs or protocol-specified significant events becomes available.

Pregnancy

In rare cases, pregnancy might occur in clinical trials. Once a female subject has been enrolled into the clinical trial, after having taken study medication, the investigator must report immediately any drug exposure during pregnancy to the sponsor. Drug exposure during pregnancy has to be reported immediately (within 24 hours or next business day

Proprietary confidential information.
© 2012 Boehringer Ingelheim International GmbH or one or more of its affiliated companies. All rights reserved.
This document may not - in full or in part - be passed on, reproduced, published or otherwise used without prior written permission.

whichever is shorter) to the defined unique entry point for SAE forms of the respective BI OPU (country-specific contact details will be provided in the ISF). The outcome of the pregnancy associated with the drug exposure during pregnancy must be followed up. In the absence of an (S)AE, only the Pregnancy Monitoring Form for Clinical Trials and not the SAE form is to be completed. The ISF will contain the Pregnancy Monitoring Form for Clinical Trials (Part A and Part B).

5.2.3 Assessment of safety and efficacy laboratory parameters

All safety and efficacy laboratory samples will be collected according to the summary of laboratory assessments based on Table 5.2.3: 1 (below). Collection of samples after a full overnight fast (nothing to eat or drink except water for at least ten hours) and before administration of BI 10773 25 mg will be required at indicated visits. Subjects should continue to take their insulin regimen as instructed by the prescribing physician.

All parameters that will be determined during the trial conduct are listed in Table 5.2.3: 2 and 5.2.3: 3. The analyses will be performed as available by a certified ~~local~~ laboratory. Lab values will be entered in eCRFs, as applicable.

Table 5.2.3: 1 Summary of laboratory assessments

Visit 1	*Visit 2*	Visit 3<br>(Clamped euglycaemia)	Visit 4<br>(Clamped Hyper-glycaemia)	Visit 8	*Visit 11*	Visit 12<br>(Clamped euglycaemia)	Visit 13<br>(Clamped Hyper-glycaemia)	*(EOT)*	Visit 15*
Day<br>-21	Day<br>-14	Day<br>1	Day<br>2	Day<br>29	Day<br>50	Day<br>57	Day<br>58	Only for early discontinued subjects (after start of therapy)	Day<br>72
Non-Fasted	*Fasted*	Non-Fasted	Non-Fasted	Non-Fasted	*Fasted*	Non-Fasted	Non-Fasted	*Fasted*	Non-Fasted*
HbA_{1c}<br>Hematology<br>Chemistry<br>TSH<br>Urine A/B<br>Urine: albumin and creatinine<br>Serum hCG	FPG<br>HbA_{1c}<br>~~GA~~ **FA**<br>Hematology<br>Chemistry<br>Lipids<br>Urine A/B	HbA_{1c}<br>~~GA~~ **FA**<br>Hematology<br>Chemistry<br>Renal panel<br>Urine A/B/C	Hematology<br>Chemistry<br>Renal panel<br>Urine A/B/C	HbA_{1c}<br>~~GA~~ **FA**<br>Hematology<br>Chemistry<br>Urine A/B	FPG<br>HbA_{1c}<br>~~GA~~ **FA**<br>Hematology<br>Chemistry<br>Lipids<br>Urine A/B	HbA_{1c}<br>~~GA~~ **FA**<br>Hematology<br>Chemistry<br>Renal panel<br>Urine A/B/C	Hematology<br>Chemistry<br>Renal panel<br>Urine A/B/C<br>Serum hCG	FPG<br>HbA_{1c}<br>~~GA~~ **FA**<br>Hematology<br>Chemistry<br>Lipids<br>Urine B<br>Serum hCG	HbA_{1c}<br>~~GA~~ **FA**<br><br>Refer to Note below
				Pre-dose PK	Pre-dose PK (same time as V8)	Pre-dose PK (same time as V8)			

Note for Visit 15: If required, other panels/tests may be repeated (fasted measurements as needed) based on clinically significant findings at Visits 11-13 or per investigator judgement.

Proprietary confidential information.
© 2012 Boehringer Ingelheim International GmbH or one or more of its affiliated companies. All rights reserved.
This document may not - in full or in part - be passed on, reproduced, published or otherwise used without prior written permission.

Table 5.2.3: 2 Safety and efficacy laboratory parameters – whole blood, serum or plasma

Hematology:

- Haematocrit
- Hemoglobin
  - Reticulocyte Count (if hemoglobin is outside normal range)
- Red Blood Cells (RBC) / Erythrocytes
- White Blood Cells (WBC) / Leukocytes
- Platelet Count / Thrombocytes
- Differential Automatic (absolute count):
  Neutrophils, Eosinophils, Basophils, Monocytes, Lymphocytes

Clinical chemistry:

- Albumin
- Alkaline phosphatase (AP)
- ALT (alanine aminotransaminase, SGPT)
- AST (aspartate aminotransaminase, SGOT)
- Bicarbonate
- Bilirubin
- Calcium
- Chloride
- Creatinine
- Creatine kinase
- Troponin
- Lactate dehydrogenase
- Lipase
- Magnesium
- Phosphate
- Potassium
- Protein total
- Sodium
- Urea
- Uric acid

Lipids:

- Cholesterol (total)
- HDL cholesterol
- LDL cholesterol
- Triglycerides

Proprietary confidential information.
© 2012 Boehringer Ingelheim International GmbH or one or more of its affiliated companies. All rights reserved.
This document may not - in full or in part - be passed on, reproduced, published or otherwise used without prior written permission.

Table 5.2.3: 2 (cont'd) Safety and efficacy laboratory parameters – whole blood, serum or plasma (Page 2 of 2)

Renal panel:

- cGMP
- Nitric oxide
- Angiotensin II (RAAS mediator)
- Angiotensinogen (RAAS mediator)
- Aldosterone (RAAS mediator)
- Plasma renin activity (RAAS mediator)
- Plasma renin concentration (RAAS mediator)
- Inulin
- Paraaminohippurate (PAH)
- Adrenaline
- Noradrenaline
- Cystatin C
- Erythropoietin

Other assessments at selected visits:

- Thyroid stimulating hormone (TSH)
- Glycated hemoglobin (HbA_{1c})
- ~~Glycated albumin (GA)~~
- **Fructosamine (FA)**
- Fasting plasma glucose (FPG)
- Human Chorionic Gonadotropin (hCG)*

*Pregnancy testing (serum) will be performed in female subjects of child bearing potential at Visit 1 and Visit 13 or EOT (based on the Summary of Laboratory Assessments table in Section 5.2.3). Additional tests may be done if locally required and per Investigator judgement.

Proprietary confidential information.
© 2012 Boehringer Ingelheim International GmbH or one or more of its affiliated companies. All rights reserved.
This document may not - in full or in part - be passed on, reproduced, published or otherwise used without prior written permission.

Table 5.2.3: 3 Safety and efficacy laboratory parameters - urine

Urinalysis:

Dipstick (A):
Immediate site assessment for leukocyte* and/or nitrite*

Routine urinalysis (B):

- Glucose
- Ketone
- Specific Gravity
- Blood
- Urine pH
- Protein
- Nitrite
- Leukocyte/Leukocyte esterase

Quantitative (C):

- Albumin **
- Creatinine **
- Sodium, glucose, urea and creatinine (24-hour)***
- cGMP
- Nitric oxide
- Prostanoids****:
  Prostaglandins E2, D2 and I
  Thromboxane B2

** at Visit 1 – albumin and creatinine will be measured
*** 24-hour measurements done only the day before Visits 3 and 12
******if applicable, the appropriate and available assay to measure the prostanoid and/or its metabolite(s) will be performed**

***Urine culture:**
A mid-stream urine sample must be sent for culture upon a positive leukocyte and/or nitrite assessment based on dipstick at the site or upon Investigator suspicion of a UTI

Albumin/creatinine ratio will be calculated.

An estimated glomerular filtration rate (eGFR) will be derived from serum creatinine values based on the MDRD formula:

eGFR (ml/min) = 175 x [Serum creatinine (umol/L)/88.4]$^{-1.154}$ x [age]$^{-0.203}$ x [0.742 if subject is female] x [1.212 if subject is of African origin]

Subjects with normal renal function (defined as eGFR ≥90 ml/min) and mild impairment of renal function (eGFR ≥60 and <90 ml/min) will be eligible for the study. Subjects with

Proprietary confidential information.
© 2012 Boehringer Ingelheim International GmbH or one or more of its affiliated companies. All rights reserved.
This document may not - in full or in part - be passed on, reproduced, published or otherwise used without prior written permission.

moderate impairment (eGFR ≥30 and <60 ml/min) and severe impairment (eGFR ≥15 and <30 ml/min) will be excluded based on Visit 1 assessments.

The estimation of renal impairment as assessed at the screening visit will be used for inclusion (i.e. eGFR must be ≥60 ml/min). The exact baseline GFR will be assessed during Visit 3 under euglycaemia and will be the basis for the stratification of subjects in the hyperfilterer or non-hyperfilterer groups. As the results of the GFR will not be available until 3-4 weeks after the completion of the renal assessments during Visit 3, recruitment may continue until a minimum of 12 hyperfilterer subjects and 12 non-hyperfilterer subjects have completed the study (Visit 13). The rational for this minimum is based on the sample size of 12 subjects per group, assuming that with a standard deviation of 5, a two-group t-test with a 0.05 two- sided significance level will provide an 80% power to detect a difference between the groups.

Criteria for hypoglycaemic events

Every episode of plasma glucose below or equal to 70 mg/dl (3.9 mmol/l) should be documented in the eCRF with the respective time and date of occurrence. Any hypoglycaemia with glucose values <54 mg/dl (<3.0 mmol/l) and all symptomatic and severe hypoglycaemias should be documented as an AE "hypoglycaemic event".

For the analysis, all hypoglycaemias will be classified according to the following criteria:

- Asymptomatic hypoglycaemia: Event not accompanied by typical symptoms of hypoglycaemia but with a measured plasma glucose concentration ≤70 mg/dl (3.9 mmol/l)
- Documented symptomatic hypoglycaemia with glucose concentration ≥54 mg/dl and ≤70 mg/dl (≥3.0 mmol/l and ≤3.9 mmol/l): Event accompanied by typical symptoms of hypoglycaemia
- Documented symptomatic hypoglycaemia with glucose concentration <54 mg/dl (<3.0 mmol/l): Event accompanied by typical symptoms of hypoglycaemia but no need for external assistance
- Severe hypoglycaemic episode: Event requiring the assistance of another person to actively administer carbohydrate, glucagon or other resuscitative actions.

Follow-up on suspicion for urinary tract infections

Subjects having a history of chronic/recurrent urinary tract infections (UTI) or genital tract infection (GTI) will be identified and this condition must to be documented under medical history / baseline condition in the eCRF. In case an acute episode of UTI or GTI is noted at screening, the subject will be screen failed and could later on be re-screened if the condition has been treated and resolved prior to re-screening.

For documentation of acute urinary tract infections during trial conduct, the following measures have to be taken:

Proprietary confidential information.
© 2012 Boehringer Ingelheim International GmbH or one or more of its affiliated companies. All rights reserved.
This document may not - in full or in part - be passed on, reproduced, published or otherwise used without prior written permission.

- In any case of suspected UTI (symptomatic or asymptomatic) a urine culture sample has to be taken and sent to the laboratory for confirmation of the diagnosis

- To be able to identify asymptomatic UTIs immediately, dipstick and urinalysis will be performed at the site at specified visits. Only in case of a positive dipstick for nitrite or leukocyte or in case of a suspicion of a UTI, a urine culture sample has to be taken and sent to the laboratory for confirmation of the diagnosis.

5.2.4 Physical examination and electrocardiogram

At Visit 1 (baseline assessment done at screening), Visit 8 and Visit 13 (end of treatment), a complete physical examination will be performed by the investigator (see Flow Chart). Documentation of, and findings from the physical examination, must be part of the source documents available at the site.

Printed paper tracings from 12-lead ECGs (I, II, III, aVR, aVL, aVF, V_1-V_6) will be collected at Visit 1 (screening) and Visit 13 (end of treatment). In the event of any cardiac symptoms (i.e. suspicion of heart rhythm disorders or cardiac ischemia), additional ECGs will be recorded. All ECGs will be evaluated (signed, dated and commented upon) by the treating physician/investigator and stored locally. Any clinically relevant changes (according to investigators judgement) in the ECG will be reported as AEs and followed up and/or treated locally until a normal or stable condition if feasible is achieved.

All ECGs performed at any time during the conduct of the trial (whether clinically relevant or routine) will be stored in the subject source notes.

5.2.5 Weight and waist circumference

Weight measurements should always be done on the same scale for each subject. In order to get comparable body weight values, it should be performed in the following way:

- after the urine sampling (weight after bladder voiding)

- shoes and coats/jackets should be taken off

- pockets should be emptied of heavy objects (i.e. keys, coins etc).

Waist circumference measurements should be made around a subject's bare midriff, after the subject exhales while standing without shoes and with both feet touching and arms hanging freely. The measuring tape should be made of a material that is not easily stretched, such as fibreglass. The tape should be placed perpendicular to the long axis of the body and horizontal to the floor and applied with sufficient tension to conform to the measurement surface.

Waist circumference should be determined by measuring the midpoint between the lowest rib and the iliac crest.

Proprietary confidential information.
© 2012 Boehringer Ingelheim International GmbH or one or more of its affiliated companies. All rights reserved.
This document may not - in full or in part - be passed on, reproduced, published or otherwise used without prior written permission.

5.3 OTHER

5.3.1 Other endpoint

5.3.1.1 The Diabetes Treatment Satisfaction Questionnaire (DTSQ)

At Visit 3, Visit 8 and Visit 12, the Diabetes Treatment Satisfaction Questionnaire (DTSQ status version) will be used as a subject reported outcome. The DTSQ in its original status form (refer to Appendix 10.1) is recommended for measuring subject satisfaction with diabetes treatment. It consists of a six-item scale assessing treatment satisfaction and two items assessing perceived frequency of hyperglycaemia and hypoglycaemia. The DTSQ is recognized by the World Health Organization (WHO) and the International Diabetes Federation (IDF) as a useful instrument in assessing outcomes of diabetes care. The DTSQs will be analyzed descriptively only – statistical tests will not be conducted.

The DTSQs is suitable for administration as a paper questionnaire. In this trial, it will be self-administered by the subject. The scores will then be transcribed into the eCRF by the Investigator (or the designated site-personnel).

A subject can self-administer the DTSQs in approximately 5 - 10 minutes, and the Investigator (or designated site-personnel) should ensure that the subject has access to a quiet area at the site where they can be left alone to record a response to all the items displayed. *In instances where a subject cannot give or decide upon a response, no response should be recorded.* The Investigator (or designated site-personnel) should check that all items have been completed by the subject, but the response to each item should not be scrutinised.

5.3.2 Other assessments

5.3.2.1 Home Blood Glucose Monitoring (HBGM)

All subjects will be provided with equipment for HBGM and supplies for use at home during the study. Instructions on proper HBGM, using a supplied BG meter, will be provided by the study staff. The subject will be asked to record the results of HBGM on supplied study HBGM logs (in a study subject diary) which will be included in the subject source document files. Only in the case of linked adverse events or of hypoglycaemia, the single value from HBGM will be recorded in the eCRF (except for the specific 8-point HBGM).

During the trial, HBGM should be performed multiple times daily as recommended by the Investigator or at any time the subject is symptomatic, i.e., experiences signs/symptoms of hyper- or hypoglycaemia. If during the trial after an overnight fast, a test result from HBGM reveals blood glucose of >13.3 mmol/l (>240 mg/dl) or <3.9 mmol/l (<70 mg/dl), the subject when feasible should contact the site immediately. The investigator should then instruct the subject to titrate their insulin regimen in order to adequately control their hyperglycaemia or hypoglycaemia. *All insulin treatment decisions must be based on blood glucose values measured using a home blood glucose meter or based on lab values obtained through the local laboratory.*

Proprietary confidential information.
© 2012 Boehringer Ingelheim International GmbH or one or more of its affiliated companies. All rights reserved.
This document may not - in full or in part - be passed on, reproduced, published or otherwise used without prior written permission.

The same blood glucose meter system will be supplied to all subjects and must be used by all subjects during the course of the study. In addition to routine daily measurements, an 8-point HBGM will be *done 1 day after Visit 2 and Visit 14 and one day prior to Visits 8 and 11 (thus subjects should start the 8-point test in the mornings of study days -13, 28, 49 and 66).*

5.3.2.2 Continuous Glucose Monitoring (CGM) Evaluation

Participants will use CGM to obtain unbiased glucose data in order to determine to what extent the administration of BI10773 as adjunctive-to-insulin in T1DM alters diurnal glycaemic patterns. Specifically, diurnal glucose exposure, variability and stability will be measured and compared within groups during the baseline period and other time points during the study treatment and follow up periods (example: first 2 weeks of treatment, 2 weeks in the middle of the treatment period, 2 last weeks of the treatment period and the post-treatment period). Changes between baseline and each study period for other parameters of glycemic variability (examples, MAGE: mean amplitude of glycaemic excursions, SD: standard deviation) will be characterized.

All subjects will be instructed to properly use the Guardian® REAL-Time Continuous Glucose Monitoring System (supplied by Medtronic of Canada Inc.). This device is commercially available with single-use disposable electrochemical sensing elements. The Guardian® REAL-Time Continuous Glucose Monitoring System allows glucose levels to be recorded for up to six days at a time (after which a sensor change is required for additional continued monitoring). Subjects will be trained to insert the sensor and perform recommended daily calibrations. Initially, and if applicable, each new glucose sensor must be calibrated after the 2-hour warm-up period, and again within 6 hours. After that, meter blood glucose values must be linked to the Guardian® system as required for proper calibration if applicable (twice daily or as needed). *Subjects will be reminded that all treatment decisions must be based on meter blood glucose values (taken several times daily) not glucose values displayed through the CGM.* Study subjects will be instructed to replace the sensor every 6 days in order to capture continuous glucose monitoring from (study day -14 to study day 72) and to verify proper sensor self-insertion and daily calibration as required.

All subjects will be required to wear the Guardian® REAL-Time Continuous Glucose Monitoring System from Visit 2 (study day -14) to Visit 15 (study day 72). Subjects will be instructed on the proper calibration of the Guardian® REAL-Time CGM System using a supplied home blood glucose meter. The investigational staff will assist each subject with the insertion of the first sensor into the abdominal area, using an insertion device (Sen-serter®); the sensor will then be connected to the MiniLink™ transmitter which will transmit continuously the interstitial concentration of glucose to the Guardian® REAL-Time CGM System using a radio frequency signal. As part of the procedures during the initial sensor insertion and transmitter set-up, subjects will remain at or near the study site until the Guardian REAL-Time Clinical device has accepted the first successful sensor calibration (approximately 2 hours). If a sensor fails two consecutive calibrations, the sensor will be replaced. Subjects may be requested to share their daily glucose profiles with the treating physician as needed in order to optimize treatment decisions. The CGM data is stored in each Guardian® REAL-Time CGM device and will be centrally uploaded to a computer (at the

Proprietary confidential information.
© 2012 Boehringer Ingelheim International GmbH or one or more of its affiliated companies. All rights reserved.
This document may not - in full or in part - be passed on, reproduced, published or otherwise used without prior written permission.

investigational site). The CGM data will then be sent via a secure internet link to the International Diabetes Center (Park Nicollet, Minneapolis, USA) for analysis.

5.3.3 Pharmacogenomic evaluation

Pharmacogenomic evaluations are not planned in this trial.

5.4 APPROPRIATENESS OF MEASUREMENTS

Measurements performed during this trial are standard or previously validated measurements and will be performed in order to monitor safety aspects and to determine BI 10773 pharmacodynamics in an appropriate way.

The scheduled measurements are appropriate to see drug induced changes in vital signs, standard laboratory values, ECG, biomarkers specific to efficacy of treatment of T1DM and renal endpoints. The primary and other endpoints are acceptable evaluations of safety and tolerability of an oral antidiabetic drug.

Therefore, the appropriateness of all measurements applied in this trial is given.

5.5 DRUG CONCENTRATION MEASUREMENTS AND PHARMACOKINETICS

5.5.1 Pharmacokinetic endpoints

Blood samples will be collected pre-dose Visits 8, 11 and 12 at the time points indicated in the Flow Chart to determine BI 10773 trough concentrations.

They will serve to characterize the following PK endpoint:

- Steady state trough concentrations of BI 10773

5.5.2 Methods of sample collection

Blood samples for pharmacokinetics will be taken from approximately 30 subjects.

The time interval to the last dose of study medication should be between 22 and 26h. For quantification of BI 10773 plasma concentrations, 3.0 ml of blood will be drawn from a forearm vein in an EDTA-anticoagulant blood drawing tube at each time point. Details of sample handling and sample logistics can be found in the ISF.

The sample should be collected within 30 minutes prior to dosing at Visit 8. The 2 other PK collections respectively at Visits 11 and 12 should be taken at **approximately** the same time as the Visit 8 sample.

Proprietary confidential information.
© 2012 Boehringer Ingelheim International GmbH or one or more of its affiliated companies. All rights reserved.
This document may not - in full or in part - be passed on, reproduced, published or otherwise used without prior written permission.

5.5.3 Analytical determinations

BI 10773 concentrations in plasma samples will be determined by a validated HPLC-MS/MS assay (high performance liquid chromatography, tandem mass spectrometry).

5.6 BIOMARKERS

No additional biomarkers will be determined in this study.

5.6.1 Endpoints based on biomarkers

Endpoints based on biomarkers are not planned in this study.

5.6.2 Methods of sample collection

Methods of sample collection are not planned in this study.

5.6.3 Analytical determinations

Analytical determinations are not planned in this study.

5.7 PHARMACODYNAMICS

No additional pharmacodynamic parameters will be determined in this study other than markers listed in previous protocol sections.

5.7.1 Pharmacodynamic endpoints

Pharmacodynamic endpoints are not planned in this study.

5.7.2 Methods of sample collection

Methods of sample collection are not planned in this study.

5.8 PHARMACOKINETIC - PHARMACODYNAMIC RELATIONSHIP

The relationship between BI 10773 trough plasma concentrations and any other biomarkers (HbA_{1c}, ~~GA~~ **FA**, FPG) will not be investigated.

Proprietary confidential information.
© 2012 Boehringer Ingelheim International GmbH or one or more of its affiliated companies. All rights reserved.
This document may not - in full or in part - be passed on, reproduced, published or otherwise used without prior written permission.

6. INVESTIGATIONAL PLAN

6.1 VISIT SCHEDULE

All trial visits should take place preferably between 7:00 AM and 11:00 AM (except Visits 3, 4, 12 and 13). If a subject mistakenly takes the trial medication on the morning of a schedule visit before attending the clinic (excluding visits Visit 3 and 4) or comes in non-fasted where a fasting condition is required, the visit should be rescheduled for another day when possible and the subject should be reminded about the expected conditions when presenting at the clinic. The rescheduled visit must take place in a short enough time-frame so that the subject has sufficient trial medication available.

All subjects are to adhere to the visit schedule as specified in the Flow Chart. If any visit has to be rescheduled, subsequent visits should follow the original visit date schedule. The trial medication packs contain sufficient medication to allow for these time windows.

6.2 DETAILS OF TRIAL PROCEDURES AT SELECTED VISITS

The Flow Chart summarizes the investigational procedures to be done at each visit. These procedures are further described below. *Adverse event (after ICF signature) and concomitant therapy assessments must be done at all clinic and telephone visits.*

6.2.1 Screening and run-in period

Screening (Visit 1)

- After the informed consent process is complete and written informed consent is obtained, the subjects will be assessed for study eligibility (Visit 1) including laboratory assessments as indicated in Section 5.2.3 (Summary of laboratory assessments).
- All other Visit 1 assessments will also be performed as summarized in the study Flow Chart.
- Subjects should be reminded to come to the next scheduled visit (Visit 2) fasting (no food or drinks, water only, for at least 10 hours).

Run-in (Visit 2)

- Visit 2 should preferably occur within 7 days of Visit 1 once it has been confirmed that the subject is eligible to continue to Visit 2. Visit 2 assessments will be performed as noted in the study Flow Chart. Labs to be done in a fasted state.
- Those subjects who qualify at screening (as determined at Visit 1), will undergo a 2 week open-label placebo run-in period (Visit 2 to Visit 3).
- Subject will be expected to take the run-in medication (placebo) once daily as described in Section 4.1.4. From the start of the run-in period, all eligible subjects will

Proprietary confidential information.
© 2012 Boehringer Ingelheim International GmbH or one or more of its affiliated companies. All rights reserved.
This document may not - in full or in part - be passed on, reproduced, published or otherwise used without prior written permission.

start CGM (using the Guardian® REAL-Time CGM System) and will continue the monitoring process until Visit 15 (last clinic visit). Subject must be instructed on the proper use of the Guardian® REAL-Time CGM System including the set up and daily calibration of the CGM components. Subjects should be contacted preferably daily or every 3-6 days during the run-in phase to ensure that they are performing study procedures and CGM calibrations adequately.

- A home blood glucose meter and all the necessary supplies will be given to subjects, including a fasting plasma glucose log (to be completed daily) and an 8-point glucose log (to be completed as specified in the study Flow Chart). All logs will be included in a subject study diary. HBGM will be done according to Section 5.3.2 and based on the Flow Chart.

- *Subjects must be reminded that all insulin dose evaluations and adjustments (basal and bolus) must be based on fingerstick blood glucose determinations using the supplied home blood glucose meter.* Subject *must not* adjust insulin therapy based on CGM values.

- Subjects will also be instructed to follow a healthy diet and exercise program during the course of the study as noted in the study Flow Chart, apart from the 7 days prior to renal assessments (at Visit 3-4 and Visit 12-13) in which case they will be prescribed a specific a high-sodium (preferably > 140 mmol/day) and moderate-protein (< 1.5 g/kg/day) diet during the 7 days preceding the start of renal assessments at baseline during Visits 3 and 4 (the diet to continue until the end of renal assessments at baseline, Visit 4) and during the 7 days preceding the start of the end of treatment renal assessments during Visits 12 and 13 (the diet to continue until the end of renal assessments).

- Subjects will also be reminded to collect their urine for 24-hours prior to the start of renal assessments during Visits 3 and 12.

6.2.2 Treatment period

Baseline renal assessments: Visits 3 and Visit 4 (prior to start of treatment on Day 3):

- Visit 3 and Visit 4 assessments will be performed as noted in the study Flow Chart and protocol. During Visit 3, subjects will undergo various renal and vascular assessments under controlled euglycaemia followed by the same assessments during Visit 4, where baseline renal and vascular measurements will be collected under controlled hyperglycaemia.

- The DTSQ should be completed by the subject at Visit 3.

- The trial medication (BI 10773) will be dispensed at the end of Visit 4.

Proprietary confidential information.
© 2012 Boehringer Ingelheim International GmbH or one or more of its affiliated companies. All rights reserved.
This document may not - in full or in part - be passed on, reproduced, published or otherwise used without prior written permission.

Treatment period: Day 3 to Visit 12 (Daily BI 10773 25 mg treatment):

- During clinic visits and phone visits, subjects will be reminded to continue the proper use of daily HBGM, log and diary maintenance and CGM (sensor change every 6 days and device calibration 2-3 times daily or as required using blood glucose values).

- Subjects will be given open-label 25 mg BI 10773 at the end of Visit 4 and will be instructed to start taking the study medication in the morning of study Day 3 and continue to take the study drug once daily in the morning (between 7-11 AM) except before clinic Visits 8, 11, 12 and 13 where they will be dosed at the clinic. During Visits 8, 11 and 12 a pre-dose PK sample is collected (prior to BI 10773 dosing). The timing of PK sample collection during Visit 11 and 12 should be **approximately** the same as during Visit 8.

- *For the next 5 days after Visit 4 (study Days 3-7), subjects must be followed up daily (phone calls) to ensure that all study safety and efficacy procedures are adequately followed. Details of daily follow up will be captured in eCRFs.*

- Subjects will be reminded at Visit 7 (phone call) about the requirement to perform an 8-point glucose profile using HBGM (study Day 28) the day before clinic Visit 8. The same will be done for the required 8-point glucose profile one day before Visit 11 (study Day 49).

- Subject will have physical exams done at Visits 1, 8 and 13 as described in the study Flow Chart and under Section 5.2.4.

- The DTSQ should be completed by the subject at Visit 8.

- During the telephone Visit 10, subjects should be reminded to come to the clinic in 1 week for clinic Visit 11 in a fasting state (no food or drinks, water only, for at least 10 hours).

- Subjects will also be instructed to continue to follow a healthy diet and exercise program during the course of the study as noted in the study Flow Chart, apart from the 7-day period prior to planned renal assessments (at Visit 12 and 13) in which case they will be prescribed a specific a high sodium / moderate protein diet. At clinic Visit 11, subjects will be reminded to follow this special diet and update their study diary accordingly.

- During clinic Visit 11, Subjects will be reminded to collect their urine for 24-hours prior to start of renal assessments at Visits 12. This should be performed at study Day 56.

Proprietary confidential information.
© 2012 Boehringer Ingelheim International GmbH or one or more of its affiliated companies. All rights reserved.
This document may not - in full or in part - be passed on, reproduced, published or otherwise used without prior written permission.

End of treatment renal assessments: Visits 12 and Visit 13 (end of treatment):

- Visit 12 and Visit 13 assessments will be performed as noted in the study Flow Chart. During Visit 12, subjects will undergo various renal and systemic vascular assessments under controlled euglycaemia following the administration of the trial drug 1.5 hours prior to start of assessments. The same assessments will be done during Visit 13 under controlled hyperglycaemia.
- The DTSQ should be completed by the subject at Visit 12
- Subjects will take their last dose of BI 10773 during clinic Visit 13

6.2.3 End of trial and follow-up period

Visits 14 and Visit 15

- Visit 14 and 15 assessments will be done at the clinic and procedures will be performed as noted in the study Flow Chart.
- Subjects will be reminded about the continued proper use of daily HBGM, log data entry and CGM (sensor change every 6 days and daily calibrations using HGBM values). Subject will return the CGM and equipments for HBGM at final clinic Visit (#15). Subjects will be reminded about the requirement to perform an 8-point glucose profile using HBGM one day after Visit 14 (study Day 66) and record the values in the study diary.
- For subjects who complete the study, with the exception of ~~GA~~ **FA** and HbA_{1c}, only abnormal lab values per the investigator's judgement will be repeated at Visit 15 (under fasting conditions if needed). Subjects will be advised ahead of time if they are required to come to Visit 15 under fasting conditions

End of Treatment Visit (only required for early discontinued subjects)

- In case of premature discontinuation from the trial (after start of BI 10773 treatment and prior to completion of Visit 13), an end of treatment (EOT) visit should be done as soon as feasible. All early discontinued subjects must perform the following safety laboratory assessments under fasted conditions: FPG, HbA_{1c}, ~~GA~~ **FA**, Hematology, Chemistry, Lipids, Urine B, hCG
- Other assessments must also be performed as noted in the study Flow Chart for early discontinued subjects under the specific EOT visit including a physical exam, vitals and an ECG. The EOT visit must be performed instead of a scheduled clinic visit for early discontinued subjects in cases where discontinuation occurs during a scheduled clinic visit. These subjects should return to the clinic for an additional follow up visit (only Visit 15 required, Visit 14 may be skipped). If feasible, early discontinued subjects should continue with CGM and HBGM until Visit 15.

Proprietary confidential information.
© 2012 Boehringer Ingelheim International GmbH or one or more of its affiliated companies. All rights reserved.
This document may not - in full or in part - be passed on, reproduced, published or otherwise used without prior written permission.

7. STATISTICAL METHODS AND DETERMINATION OF SAMPLE SIZE

7.1 STATISTICAL DESIGN - MODEL

This is an open-label, single centre pilot study to characterize: 1) the impact of BI 10773 on renal hyperfiltration in subjects with T1DM under conditions of controlled euglycaemia and hyperglycaemia and 2) the safety and efficacy of BI 10773-treatment as adjunctive-to-insulin therapy in these subjects.

Subjects who successfully complete the open-label placebo run-in period and meet the inclusion/exclusion criteria will be entered in the study and receive BI 10773 25 mg QD for 8 weeks. The subjects will be stratified into 2 groups based on their baseline GFR measured under a clamped euglycaemia condition at Visit 3 assessed by inulin measurements. The non-hyperfilterers will be subjects with a measured GFR of $\geq$ 60 ml/min/1.73m^2 to < 135 ml/min/1.73m^2 and hyperfilterers will be subjects with a measured GFR $\geq$ 135 ml/min/1.73m^2.

Table 7.1: 1 below describes the population, clamped condition, baseline and treatment assessments. Renal data (e.g., GFR) will be collected on days 1, 2, 57 and 58 for Hyperfilterer and Non-Hyperfilterer subjects.

Table 7.1: 1 Summary of populations, clamped conditions, baseline and treatment assessments

	**Baseline**		**After 8 weeks of treatment with BI 10773**	
Population	***Clamped Condition***		***Clamped Condition***	
	Day 1	***Day 2***	***Day 57***	***Day 58***
Hyperfilterers	Euglycaemia	Hyperglycaemia	Euglycaemia	Hyperglycaemia
Non-Hyperfilterers				

Objectives

The primary objective of this trial is to determine the impact of 8 weeks of treatment with BI 10773 (25 mg QD) on renal hyperfiltration in subjects with type 1 diabetes mellitus under conditions of controlled euglycaemia and hyperglycaemia.

The secondary objective of this trial is to characterize the safety and efficacy of BI 10773 (25 mg QD) in subjects with T1DM.

Primary endpoint

The primary endpoint is described in detail in Section 5.1.

Proprietary confidential information.
© 2012 Boehringer Ingelheim International GmbH or one or more of its affiliated companies. All rights reserved.
This document may not - in full or in part - be passed on, reproduced, published or otherwise used without prior written permission.

Secondary endpoints

There are no secondary endpoints specified for this trial.

Other endpoints

The other endpoints are described in detail in Section 5.1.

Safety endpoints

The safety endpoints are described in detail in Section 5.2.

Baseline (study baseline)

The baseline laboratory assessments are defined below:

Visit 2 (Day -14, Fasted):

- FPG
- Lipids

Visit 3 (Day 1, Non-Fasted):

- Renal panel (clamped euglycaemia condition)
- HbA_{1c}
- Hematology
- Chemistry
- Urine A/B/C
- ~~GA~~ **FA**

Visit 4 (Day 2, Non-Fasted):

- Renal panel (clamped hyperglycaemia condition)

For symptomatic hypoglycaemic events the pre-treatment period is defined as the 2 week run-in period. For time spent in CGM-defined hypoglycaemia, the pre-treatment period is defined as the 2 week of CGM assessment during the run-in phase. Visit 3 renal measurements and Visit 4 renal measurements are respectively baseline measurements for Visit 12 and 13.

7.2 NULL AND ALTERNATIVE HYPOTHESES

The following primary hypotheses will be tested using a one-tailed test at an alpha level of 0.025. The basic hypothesis to be tested is:

H_0: $\mu_d = 0$ vs. H_1: $\mu_d > 0$,

where μ_d is the mean change from baseline response after 8 weeks of treatment of BI 10773.

Since this is an exploratory study no adjustment in the alpha will be necessary.

Proprietary confidential information.
© 2012 Boehringer Ingelheim International GmbH or one or more of its affiliated companies. All rights reserved.
This document may not - in full or in part - be passed on, reproduced, published or otherwise used without prior written permission.

Hypothesis 1 under hyperglycaemia:

1. H_{01}: For hyperfilterer subjects, there is no change in GFR under the clamped hyperglycaemia condition after 8 weeks of treatment with BI 10773 (25 mg QD).

2. H_{11}: For hyperfilterer subjects, there is a decrease in GFR under the clamped hyperglycaemia condition after 8 weeks of treatment with BI 10773 (25 mg QD).

Hypothesis 2 under euglycaemia:

3. H_{02}: For hyperfilterer subjects, there is no change in GFR under the clamped euglycaemia condition after 8 weeks of treatment with BI 10773 (25 mg QD).

4. H_{12}: For hyperfilterer subjects, there is a decrease in GFR under the clamped euglycaemia condition after 8 weeks of treatment with BI 10773 (25 mg QD).

7.3 PLANNED ANALYSES

All individual values will be listed and standard statistical parameters (number of non-missing values, mean, standard deviation, median, quartiles and range) or frequency tables will be calculated separately for each treatment group (including the respective totals). The data recorded will be used to check whether the study protocol has been adhered to (e.g., inclusion and exclusion criteria, visit schedules etc.). Data from subjects that withdraw after the screening period and prior to Visit 3 will not be reported.

The statistical analysis will be based on the following populations:

- Treated Set (TS): The treated set (TS) will consist of all subjects who were treated with at least one dose of study drug.

- Full Analysis Set for Renal (FAS_RENAL): The full analysis set (FAS_RENAL) will consist of all subjects who were treated with study drug, have a baseline measurement and an evaluable post-dosing renal data under the clamped hyperglycaemia condition (Visit 13) for the primary endpoint.

- Full Analysis Set for Diabetes Mellitus (FAS_T1DM): The full analysis set (FAS_T1DM) will consist of all subjects who were treated with study drug, have a baseline measurement and an evaluable post-dosing diabetes mellitus data for the other endpoints.

7.3.1 Primary analysis

Paired t-tests will be performed to test the difference in the GFR response between baseline and after treatment of BI 10773 (25 mg QD) in hyperfilterer subjects under separate

Proprietary confidential information.
© 2012 Boehringer Ingelheim International GmbH or one or more of its affiliated companies. All rights reserved.
This document may not - in full or in part - be passed on, reproduced, published or otherwise used without prior written permission.

conditions of controlled euglycaemia and hyperglycaemia. The primary analysis will be performed on the FAS_RENAL.

7.3.2 Secondary analyses

7.3.2.1 Secondary analyses

The secondary analyses will focus on additional between-group and within-group comparisons of the primary endpoint. The analyses will be performed on the FAS_RENAL.

Since the hyperfilterer and non-hyperfilterer subjects were not randomized into the trial, the two groups may not be comparable. Thus, caution should be used interpreting the results of the between-group analyses.

<u>Within group comparisons:</u>

Paired t-tests will be performed to test the difference in the GFR response between baseline and after treatment of BI 10773 (25 mg QD) in non-hyperfilterer subjects under separate conditions of controlled euglycaemia and hyperglycaemia.

Paired t-tests will be performed to test the difference in the GFR response between baseline and after treatment of BI 10773 (25 mg QD) in all subjects under separate conditions of controlled euglycaemia and hyperglycaemia.

<u>Between-group comparisons (hyperfilterer vs non-hyperfilterer):</u>

Analysis of covariance (ANCOVA) will be performed on the primary endpoint to assess the difference between hyperfilterer versus non-hyperfilterer subjects under separate conditions of controlled euglycaemia and hyperglycaemia.

The model will include the baseline GFR and group (i.e., hyperfilterer vs. non-hyperfilterer) as independent parameters. In the event of significant results for the group covariate, adjusted means will be reported. It is hypothesized that there is a difference in the mean change of the GFR between the hyperfilterer subjects and non-hyperfilterer subjects.

In addition, the interaction of the primary endpoint will be assessed as a sensitivity analysis.

7.3.2.2 Other analyses

<u>Other exploratory renal analyses</u>
For the other exploratory renal endpoints, between-group and within-group comparisons will be performed. The analysis will be performed on the FAS_RENAL.

<u>Within group comparisons:</u>
Paired t-tests will be performed to test the difference in the response between baseline and after treatment of BI 10773 (25 mg QD) in hyperfilterer subjects under separate conditions of controlled euglycaemia and hyperglycaemia.

Proprietary confidential information.
© 2012 Boehringer Ingelheim International GmbH or one or more of its affiliated companies. All rights reserved.
This document may not - in full or in part - be passed on, reproduced, published or otherwise used without prior written permission.

Paired t-tests will be performed to test the difference in the response between baseline and after treatment of BI 10773 (25 mg QD) in non-hyperfilterer subjects under separate conditions of controlled euglycaemia and hyperglycaemia.

Paired t-tests will be performed to test the difference in the response between baseline and after treatment of BI 10773 (25 mg QD) in all subjects under separate conditions of controlled euglycaemia and hyperglycaemia.

The following analyses will be performed:

- Difference in the renal hemodynamic function (ERPF, RBF, FF, RVR) response between baseline and after treatment of BI 10773 (25 mg QD).
- Difference in the systemic hemodynamic function (MAP and arterial stiffness) response between baseline and after treatment of BI 10773 (25 mg QD).
- Difference in the circulating levels of mediators involved in RAAS activation and markers of sympathetic activity response between baseline and after treatment of BI 10773 (25 mg QD).
- Difference in the markers of glomerular and tubular integrity (nitric oxide, prostanoids and albumin excretion) response between baseline and after treatment of BI 10773 (25 mg QD).

<u>Between-group comparisons (hyperfilterer vs non-hyperfilterer):</u>
Analysis of covariance (ANCOVA) will be performed on the exploratory renal endpoints to assess the difference between hyperfilterer versus non-hyperfilterer subjects under separate conditions of controlled euglycaemia and hyperglycaemia.

The model will include the renal endpoint at baseline and group (i.e., hyperfilterer vs. non-hyperfilterer) as independent parameters. In the event of significant results for the group covariate, adjusted means will be reported. It is hypothesized that there is a difference in the mean change of the renal endpoint between the hyperfilterer subjects and non-hyperfilterer subjects.

<u>Other exploratory diabetes analyses</u>
Descriptive statistics will be used to summarize and evaluate the other diabetes-related endpoints. In addition, analyses will be performed to evaluate the follow-up period (Visits 14 and 15). These analyses will be performed on the FAS_T1DM.

Proprietary confidential information.
© 2012 Boehringer Ingelheim International GmbH or one or more of its affiliated companies. All rights reserved.
This document may not - in full or in part - be passed on, reproduced, published or otherwise used without prior written permission.

Definition of responder for HbA$_{1c}$, hypoglycaemia and insulin requirements:

Responder classification will be performed based on the change in HbA$_{1c}$, hypoglycaemia and insulin requirements from baseline. The responder criterion will be defined as a composite of the following parameters:

- HbA$_{1c}$ change:
  - Improvement: Defined as ≥ 0.3% lowering relative to baseline
  - No change: Defined < 0.3% lowering to > 0.3% increase relative to baseline
  - Worsening: Defined as ≥ 0.3% increase relative to baseline
- Hypoglycaemia change:
  - Improvement: Defined as 30% lowering relative to baseline
  - No change: Defined < 30% lowering to > 30% increase relative to baseline
  - Worsening: Defined as ≥ 30% increase relative to baseline
- Insulin requirement change:
  - Improvement: Defined as 20% lowering relative to baseline
  - No change: Defined < 20% lowering to > 20% increase relative to baseline
  - Worsening: Defined as ≥ 20% increase relative to baseline

Details of responder classification are provided below (Figure 7.3.2.2: 1):

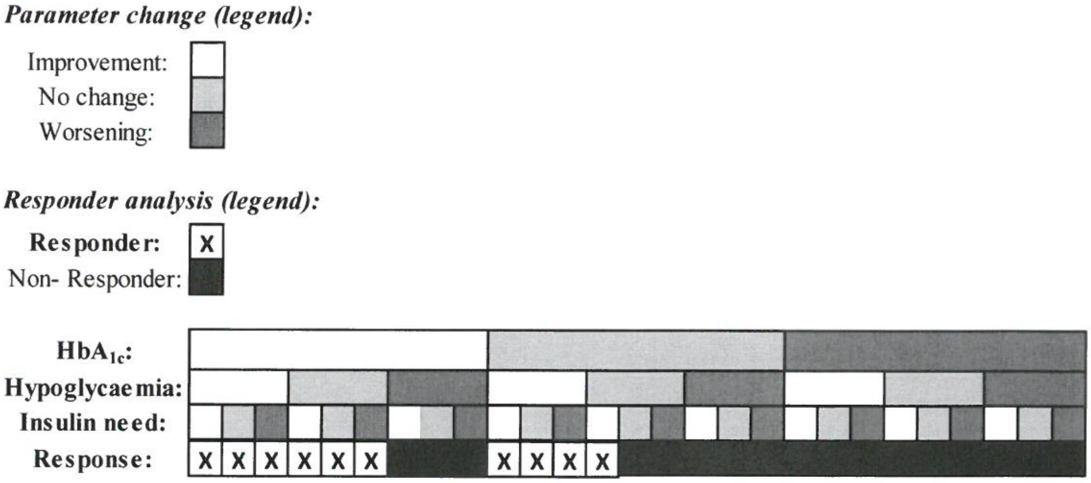

Per the above classification, a responder (X) is defined as a subject with:

- An improved HbA$_{1c}$ and an improved or unchanged hypoglycaemia, irrespective of insulin changes
- An unchanged HbA$_{1c}$ with an improved hypoglycaemia, irrespective of insulin changes
- An unchanged HbA$_{1c}$ and an unchanged hypoglycaemia with improved insulin requirements

Figure 7.3.2.2: 1 Summary of responder classification

Proprietary confidential information.
© 2012 Boehringer Ingelheim International GmbH or one or more of its affiliated companies. All rights reserved.
This document may not - in full or in part - be passed on, reproduced, published or otherwise used without prior written permission.

Continuous glucose monitoring analysis

Some glycaemia-related endpoints will be determined using ambulatory glucose profile (AGP) analysis of CGM data. AGP analysis utilizes graphic displays and statistical models to depict glucose exposure, variability and stability for periods ranging from 1 to 90 days of continuous monitoring. CGM values are collected and analyzed on a single or modal day basis without regard to a date. AGP is comprised of five time-series smoothed curves representing the 10th, 25th, 50th (median), 75th and 90th frequency percentiles for CGM values. The Area Under the median Curve (AUC) represents diurnal glucose exposure for the period under investigation. Total AUC is calculated as the sum of 24 hourly medians and reported as mg/dL/24hr. For this study, each AGP will represent a minimum of a 14-day period of continuous glucose monitoring. CGM is diurnal and hence allows for characterization of daytime vs nocturnal glucose exposures. Using the subject's home glucose logs and by visualization of AGP, the periods of waking/sleeping can be characterized and related exposure can be calculated as the sum of hourly median values during these time segments. Since activity (sleeping / waking) varies from period to period, for comparability, all values are normalized (mg/dL/hr) to reflect the average hourly glucose exposure during each study period. Postprandial glucose exposure can be measured in the same manner. The time of meal initiation and the three to four hour period following are generally used to measure postprandial glucose excursions. Total area under the postprandial median curve, as well as, normalized values (mg/dL/hr) are automatically calculated.

The change from baseline to end of study in the measurement of the inter-quartile range (IQR, mg/dL), a continuous CGM variability marker, will be an ~~important~~ exploratory parameter. Other exploratory AGP glycaemia endpoints involve comparisons between treatment groups for measurements of glucose exposure (area under the median interstitial glucose curve [AUC, mg/dL/24hr]) and glucose stability (pooled rate of change in the median curve, average moment-to-moment change in interstitial glucose, mg/dL/hr), % time in hyperglycaemia (>180 mg/dL or >10 mmol/l), % time in hypoglycaemia (<60 mg/dL or <3.3 mmol/l) and proportion of CGM readings within 70-140 mg/dL (3.9-7.8 mmol/l). Additional glycaemia measurers include MAGE and average durations of the hyperglycaemic and hypoglycaemic episodes, mean values from HBGM and changes in the proportion of subjects within clinical target categories. Parameter changes from the baseline to end of study will be analyzed within the group.

HBGM/ CGM comparison

AGPs produced by CGM data which coincide with periods of 8-point HBGM will be compared for to the following parameters: glucose exposure, glucose stability, glucose variability, time lag and relative difference in each value.

7.3.3 Safety analyses

Safety analysis will be performed in accordance with BI standards and will be based on the treated set population, i.e., all treated subjects. Descriptive statistics will be used to summarize and evaluate the safety endpoints.

Proprietary confidential information.
© 2012 Boehringer Ingelheim International GmbH or one or more of its affiliated companies. All rights reserved.
This document may not - in full or in part - be passed on, reproduced, published or otherwise used without prior written permission.

7.3.4 Interim analyses

An interim analysis is not planned for this trial.

7.3.5 Pharmacokinetic analyses

Pharmacokinetic data will be collected for this study as defined in Section 5.5.1.

7.3.6 Pharmacodynamic analyses

Pharmacodynamic data will not be collected for this study.

7.3.7 Pharmacogenomic analyses

Pharmacogenomic data will not be collected for this study.

7.4 HANDLING OF MISSING DATA

No special computations (e.g., interpolation, last value carried forward or similar) will be performed for the missing data.

7.5 RANDOMISATION

This is an open-label single centre pilot study of BI 10773. There is no randomization for this study.

7.6 DETERMINATION OF SAMPLE SIZE

For this trial, between-group analysis and within-group analysis will be performed. The sample size calculations are based on the between-group analysis. Listed below in Table 7.6: 1 are the sample size calculations based on a two-group t-test (two-sided, α=0.05), which looks at the difference between the hyperfilterer and non-hyperfilterer groups.

With the sample size of 12 subjects per group and assuming that the standard deviation is 5, a two-group t-test with a 0.05 two-sided significance level will have 80% power to detect a difference in the means of 6 (i.e., the difference between group 1 (Hyperfilterer) mean and Group 2 (Non-hyperfilterer) mean). If the standard deviation is increased to 6, a sample size of 17 subjects per group would be required. As such, a minimum of 12 subjects per group will need to complete the trial (Visit 13) and therefore approximately 30 subjects will need to be entered to achieve the sample size requirements.

Proprietary confidential information.
© 2012 Boehringer Ingelheim International GmbH or one or more of its affiliated companies. All rights reserved.
This document may not - in full or in part - be passed on, reproduced, published or otherwise used without prior written permission.

Table 7.6: 1 Sample size calculations based on a two-group t-test (two-sided, α=0.05)

Mean Change for Hyperfilterer Group	Mean Change for Non-hyperfilterer Group	Difference between the groups	Standard Deviation	Power (%)	N per group
8	5	3	3.5	80	23
8	5	3	5	80	45
8	5	3	6	80	64
10	5	5	3.5	80	9
10	5	5	5	80	17
10	5	5	6	80	24
11	5	6	3.5	80	7
11	5	6	5	80	12
11	5	6	6	80	17
11	5	6	8	80	29

The sample size estimations were performed using the MTT0-1 routine from the commercial software nQuery Advisor® version 6.01 (Statistical Solutions, Ltd., Cork, Ireland, [R99-2294]).

Proprietary confidential information.
© 2012 Boehringer Ingelheim International GmbH or one or more of its affiliated companies. All rights reserved.
This document may not - in full or in part - be passed on, reproduced, published or otherwise used without prior written permission.

8. INFORMED CONSENT, DATA PROTECTION, TRIAL RECORDS

The trial will be carried out in compliance with the protocol, the principles laid down in the Declaration of Helsinki, version as of October 1996 (as long as local laws do not require to follow other versions), in accordance with the ICH Harmonised Tripartite Guideline for Good Clinical Practice (GCP) and relevant BI SOPs. Standard medical care (prophylactic, diagnostic and therapeutic procedures) remains in the responsibility of the treating physician of the patient.

The investigator should inform the sponsor immediately of any urgent safety measures taken to protect the study patients against any immediate hazard, and also of any serious breaches of the protocol/ICH GCP.

The rights of the investigator and of the sponsor with regard to publication of the results of this trial are described in the investigator contract. As a general rule, no trial results should be published prior to finalisation of the CTR.

Insurance Cover: The terms and conditions of the insurance cover are made available to the investigator and the patients via documentation in the ISF.

8.1 STUDY APPROVAL, PATIENT INFORMATION, AND INFORMED CONSENT

This trial will be initiated only after all required legal documentation has been reviewed and approved by the respective IRB / IEC and CA according to national and international regulations. The same applies for the implementation of changes introduced by amendments.

Prior to patient participation in the trial, written informed consent must be obtained from each patient (or the patient's legally accepted representative) according to ICH GCP and to the regulatory and legal requirements of the participating country. Each signature must be personally dated by each signatory and the informed consent and any additional patient-information form retained by the investigator as part of the trial records. A signed copy of the informed consent and any additional patient information must be given to each patient or the patient's legally accepted representative.

The patient must be informed that his / her personal trial-related data will be used by BI in accordance with the local data protection law. The level of disclosure must also be explained to the patient.

The patient must be informed that his / her medical records may be examined by authorised monitors (CML / CRA) or Clinical Quality Assurance auditors appointed by BI, by appropriate IRB / IEC members, and by inspectors from regulatory authorities.

Proprietary confidential information.
© 2012 Boehringer Ingelheim International GmbH or one or more of its affiliated companies. All rights reserved.
This document may not - in full or in part - be passed on, reproduced, published or otherwise used without prior written permission.

8.2 DATA QUALITY ASSURANCE

A quality assurance audit/inspection of this trial may be conducted by the sponsor or sponsor's designees or by IRBs / IECs or by regulatory authorities. The quality assurance auditor will have access to all medical records, the investigator's trial-related files and correspondence, and the informed consent documentation of this clinical trial.

8.3 RECORDS

Case Report Forms (CRFs) for individual patients will be provided by the sponsor, either on paper or via remote data capture. See Section 4.1.5.2 for rules about emergency code breaks. For drug accountability, refer to Section 4.1.8.

8.3.1 Source documents

Source documents provide evidence for the existence of the patient and substantiate the integrity of the data collected. Source documents are filed at the investigator's site.

Data entered in the eCRFs that are transcribed from source documents must be consistent with the source documents or the discrepancies must be explained. The investigator may need to request previous medical records or transfer records, depending on the trial; also current medical records must be available.

For eCRFs all data must be derived from source documents.

8.3.2 Direct access to source data and documents

The investigator / institution will permit trial-related monitoring, audits, IRB / IEC review and regulatory inspection, providing direct access to all related source data / documents. CRFs/eCRFs and all source documents, including progress notes and copies of laboratory and medical test results must be available at all times for review by the sponsor's clinical trial monitor, auditor and inspection by health authorities (e.g. FDA). The Clinical Research Associate (CRA) / on site monitor and auditor may review all CRFs/eCRFs, and written informed consents. The accuracy of the data will be verified by reviewing the documents described in Section 8.3.1.

8.4 LISTEDNESS AND EXPEDITED REPORTING OF ADVERSE EVENTS

8.4.1 Listedness

To fulfil the regulatory requirements for expedited safety reporting, the sponsor evaluates whether a particular adverse event is "listed", i.e. is a known side effect of the drug or not. Therefore a unique reference document for the evaluation of listedness needs to be provided. For BI 10773 this is the current version of the Investigator's Brochure. The current versions of these reference documents are to be provided in the ISF. No AEs are classified as listed for matching placebo, study design, or invasive procedures.

Proprietary confidential information.
© 2012 Boehringer Ingelheim International GmbH or one or more of its affiliated companies. All rights reserved.
This document may not - in full or in part - be passed on, reproduced, published or otherwise used without prior written permission.

8.4.2 Expedited reporting to health authorities and IECs/IRBs

Expedited reporting of serious adverse events, e.g. suspected unexpected serious adverse reactions (SUSARs) to health authorities and IECs/IRBs, will be done according to local regulatory requirements. Further details regarding this reporting procedure are provided in the Investigator Site File.

8.5 STATEMENT OF CONFIDENTIALITY

Individual patient medical information obtained as a result of this trial is considered confidential and disclosure to third parties is prohibited with the exceptions noted below. Patient confidentiality will be ensured by using patient identification code numbers.

Treatment data may be given to the patient's personal physician or to other appropriate medical personnel responsible for the patient's welfare. Data generated as a result of the trial need to be available for inspection on request by the participating physicians, the sponsor's representatives, by the IRB / IEC and the regulatory authorities i.e. the CA.

Proprietary confidential information.
© 2012 Boehringer Ingelheim International GmbH or one or more of its affiliated companies. All rights reserved.
This document may not - in full or in part - be passed on, reproduced, published or otherwise used without prior written permission.

9. REFERENCES

9.1 PUBLISHED REFERENCES

R05-0939 Wright EM, Turk E. The sodium/glucose cotransport family SLC5. Pfluegers Arch 2004;447(5):510-518.

R10-0703 Rahmoune H, Thompson PW, Ward JM, Smith CD, Hong G, Brown J. Glucose transporters in human renal proximal tubular cells isolated from the urine of patients with non-insulin-dependent diabetes. Diabetes 2005;54(12):3427-3434.

R10-6364 Cherney DZI, Scholey JW, Nasrallah R, Dekker MG, Slorach C, Bradley TJ, Hebert RL, Sochett EB, Miller JA. Renal hemodynamic effect of cyclooxygenase 2 inhibition in young men and women with uncomplicated type 1 diabetes mellitus. Am J Physiol 2008;294:F1336-F1341.

R10-6365 Cherney DZI, Miller JA, Scholey JW, Bradley TJ, Slorach C, Curtis JR, Dekker MG, Nasrallah R, Hebert RL, Sochett EB. The effect of cyclooxygenase-2 inhibition on renal hemodynamic function in humans with type 1 diabetes. Diabetes 2008;57:688-695.

R10-6366 Cherney DZI, Lai V, Scholey JW, Miller JA, Zinman B, Reich HN. Effect of direct renin inhibition on renal hemodynamic function, arterial stiffness, and endothelial function in humans with uncomplicated type 1 diabetes: a pilot study. Diabetes Care 2010;33(2):361-365.

R10-6367 Cherney DZI, Konvalinka A, Zinman B, Diamandis EP, Soosalipillai A, Reich H, Lorraine J, Lai V, Scholey JW, Miller JA. Effect of protein kinase Cbeta inhibition on renal hemodynamic function and urinary biomarkers in humans with type 1 diabetes: a pilot study. Diabetes Care 2009;32(1):91-93.

R10-6368 Sochett EB, Cherney DZI, Curtis JR, Dekker MG, Scholey JW, Miller JA. Impact of renin angiotensin system modulation on the hyperfiltration state in type 1 diabetes. J Am Soc Nephrol 2006;17:1703-1709.

R10-6369 Fatourechi MM, Kudva YC, Murad MH, Elamin MB, Tabini CC, Montori VM. Hypoglycemia with intensive insulin therapy: a systematic review and meta-analyses of randomized trials of continuous subcutaneous insulin infusion versus multiple daily injections. J Clin Endocrinol Metab 2009;94(3):729-740.

R10-6370 Bluestone JA, Herold K, Eisenbarth G. Genetics, pathogenesis and clinical interventions in type 1 diabetes. Nature 2010;464:1293-1300.

Proprietary confidential information.
© 2012 Boehringer Ingelheim International GmbH or one or more of its affiliated companies. All rights reserved.
This document may not - in full or in part - be passed on, reproduced, published or otherwise used without prior written permission.

R10-6371 Bergenstal RM, Tamborlane WV, Ahmann A, Buse JB, Dailey G, Davis SN, Joyce C, Peoples T, Perkins BA, Welsh JB, Willi SM, Wood MA, STAR 3 Study Group. Effectiveness of sensor-augmented insulin-pump therapy in type 1 diabetes. N Engl J Med 2010;363(4):311-320.

R10-6374 Cherney DZI, Miller JA, Scholey JW, Nasrallah R, Hebert RL, Dekker MG, Slorach C, Sochett EB, Bradley TJ. Renal hyperfiltration is a determinant of endothelial function responses to cyclooxygenase 2 inhibition in type 1 diabetes. Diabetes Care 2010;33(6):1344-1346.

R10-6375 Cherney DZI, Sochett EB, Lai V, Dekker MG, Slorach C, Scholey JW, Bradley TJ. Renal hyperfiltration and arterial stiffness in humans with uncomplicated type 1 diabetes. Diabetes Care 2010;33(9):2068-2070.

R10-6376 Cramer JA. A systematic review of adherence with medications for diabetes. Diabetes Care 2004;27(5):1218-1224.

R10-6377 Thorn LM, Forsblom C, Fagerudd J, Thomas MC, Pettersson-Fernholm K, Saraheimo M, Waden J, Ronnback M, Rosengard-Barlund M, Bjorkesten CG af, Taskinen MR, Groop PH, FinnDiane Study Group. Metabolic syndrome in type 1 diabetes: association with diabetic nephropathy and glycemic control (the FinnDiane study). Diabetes Care 2005;28(8):2019-2024.

R10-6380 Mustata S, Chan C, Lai V, Miller JA. Impact of an exercise program on arterial stiffness and insulin resistance in hemodialysis patients. J Am Soc Nephrol 2004;15(10):2713-2718.

R10-6572 Mogensen CE. Maximum tubular reabsorption capacity for glucose and renal hemodynamics during rapid hypertonic glucose infusion in normal and diabetic subjects. Scand J Clin Lab Invest 1971;28(1):101-109.

R11-0072 Cherney DZI, Reich HN, Miller JA, Lai V, Zinman B, Dekker MG, Bradley TJ, Scholey JW, Sochett EB. Age is a determinant of acute hemodynamic responses to hyperglycemia and angiotensin II in humans with uncomplicated type 1 diabetes mellitus. Am J Physiol 2010;299(1):R206-R214.

R99-2294 Elashoff JD. nQuery Advisor version 3.0 user's guide. Los Angeles: Statistical Solutions 1999

9.2 UNPUBLISHED REFERENCES

Unpublished references are not included in this protocol.

Proprietary confidential information.
© 2012 Boehringer Ingelheim International GmbH or one or more of its affiliated companies. All rights reserved.
This document may not - in full or in part - be passed on, reproduced, published or otherwise used without prior written permission.

10. APPENDICES

10.1 DTSQs

Diabetes Treatment Satisfaction Questionnaire: DTSQs

The following questions are concerned with the treatment for your diabetes (including insulin, tablets and/or diet) and your experience over the past few weeks. Please answer each question by circling a number on each of the scales.

1. How satisfied are you with your current treatment?

very satisfied	6	5	4	3	2	1	0	very dissatisfied

2. How often have you felt that your blood sugars have been unacceptably high recently?

most of the time	6	5	4	3	2	1	0	none of the time

3. How often have you felt that your blood sugars have been unacceptably low recently?

most of the time	6	5	4	3	2	1	0	none of the time

4. How convenient have you found your treatment to be recently?

very convenient	6	5	4	3	2	1	0	very inconvenient

5. How flexible have you found your treatment to be recently?

very flexible	6	5	4	3	2	1	0	very inflexible

6. How satisfied are you with your understanding of your diabetes?

very satisfied	6	5	4	3	2	1	0	very dissatisfied

7. Would you recommend this form of treatment to someone else with your kind of diabetes?

Yes, I would definitely recommend the treatment	6	5	4	3	2	1	0	No, I would definitely not recommend the treatment

8. How satisfied would you be to continue with your present form of treatment?

very satisfied	6	5	4	3	2	1	0	very dissatisfied

Please make sure that you have circled one number on each of the scales.

DTSQs © Prof Clare Bradley 9/93 English for Canada 20.1.06 (from Standard UK English rev. 7/94)
Health Psychology Research, Dept of Psychology, Royal Holloway, University of London, Egham, Surrey, TW20 0EX, UK.

Proprietary confidential information.
© 2012 Boehringer Ingelheim International GmbH or one or more of its affiliated companies. All rights reserved.
This document may not - in full or in part - be passed on, reproduced, published or otherwise used without prior written permission.

11. DESCRIPTION OF GLOBAL AMENDMENTS

**Number of global amendment**		2
**Date of CTP revision**		20 Aug 2012
**BI Trial number**		1245.46
**BI Investigational Product(s)**		BI 10773
**Title of protocol**		An open-label 8-week adjunctive-to-insulin and renal mechanistic pilot trial of BI 10773 in type 1 diabetes mellitus (the ATIRMA trial)
**To be implemented only after approval of the IRB/IEC/Competent Authorities**		☐
**To be implemented immediately in order to eliminate hazard – IRB / IEC / Competent Authority to be notified of change with request for approval**		☐
**Can be implemented without IRB/IEC/ Competent Authority approval as changes involve logistical or administrative aspects only**		☒
**Section to be changed**		Title page
**Description of change**		Change 1: Revised Doc. No. to comply with the new protocol version number.<br>Change 2: Addition of a new protocol date and version number.
**Rationale for change**		To reflect the new revised protocol date and version number.
**Section to be changed**		Synopsis
**Description of change**		Revised protocol date and revision date.
**Rationale for change**		Clarification
**Section to be changed**		Throughout entire protocol
**Description of change**		Glycated albumin (GA) test name revised to fructosamine (FA). FA also added to the

Proprietary confidential information.
© 2012 Boehringer Ingelheim International GmbH or one or more of its affiliated companies. All rights reserved.
This document may not - in full or in part - be passed on, reproduced, published or otherwise used without prior written permission.

		Abbreviations section.
**Rationale for change**		Clarification
**Section to be changed**		4.2 CONCOMITANT THERAPY, RESTRICTIONS, AND RESCUE TREATMENT
**Description of change**		The words “and rescue treatment” added to the heading of this section to comply with company standards for protocol format structure/headings.
**Rationale for change**		Clarification
**Section to be changed**		5.1.2.5 Glycated hemoglobin (HbA1c) and Fructosamine (FA)
**Description of change**		Reference to NGSP Level 1 certification removed.
**Rationale for change**		Clarification
**Section to be changed**		Flow Chart footnotes, section 5.5.2 Methods of sample collection and section 6.2.2 Treatment period
**Description of change**		The word “approximately” added.
**Rationale for change**		Clarification
**Section to be changed**		Table 5.2.3: 3 Safety and efficacy laboratory parameters - urine
**Description of change**		Clarifications added for planned prostanoid assays
**Rationale for change**		Clarification

**Number of global amendment**		1
**Date of CTP revision**		28 Nov 2011
**BI Trial number**		1245.46
**BI Investigational Product(s)**		BI 10773
**Title of protocol**		An open-label 8-week adjunctive-to-insulin and renal mechanistic pilot trial of BI 10773 in type 1 diabetes mellitus (the ATIRMA trial)
**To be implemented only after approval of the IRB/IEC/Competent Authorities**		☐
**To be implemented immediately in order to**		☐

Proprietary confidential information.
© 2012 Boehringer Ingelheim International GmbH or one or more of its affiliated companies. All rights reserved.
This document may not - in full or in part - be passed on, reproduced, published or otherwise used without prior written permission.

**eliminate hazard – IRB / IEC / Competent Authority to be notified of change with request for approval**		
**Can be implemented without IRB/IEC/ Competent Authority approval as changes involve logistical or administrative aspects only**		☒
**Section to be changed**		Title page
**Description of change**		Change 1: Revised Doc. No. to comply with the new protocol version number.<br>Change 2: Addition of a new protocol date and version number.
**Rationale for change**		To reflect the new revised protocol date and version number.
**Section to be changed**		Synopsis
**Description of change**		Change 1: Addition of a protocol revision date.<br>Change 2: Clarification to BMI units in inclusion # 5.
**Rationale for change**		Clarifications
**Section to be changed**		Flow Chart and footnotes H, L and K
**Description of change**		Change 1: The word "urine" deleted from the Pregnancy Test row in the flow chart and in corresponding footnote H as a serum assessment will be done.<br>Change 2: The name of the 24-hour "UGE" was changed to "urine collection" for clarity; this assessment will measure sodium, urea, creatinine in addition to glucose. Footnote L corrected accordingly.<br>Change 3: Within the scheduled lab table under footnote K, the type of hCG assessment under Visits 1, 13 and EOT was clarified. The preferred time point of Visit 1 was also corrected from -27 days to -21 days.
**Rationale for change**		Clarification
**Section to be changed**		Abbreviations
**Description of change**		Addition of DMC (Data-Monitoring Committee) and hCG (Human Chorionic Gonadotropin)

Proprietary confidential information.
© 2012 Boehringer Ingelheim International GmbH or one or more of its affiliated companies. All rights reserved.
This document may not - in full or in part - be passed on, reproduced, published or otherwise used without prior written permission.

**Rationale for change**		Clarification
**Section to be changed**		3.1.1 Administrative structure of the trial
**Description of change**		Addition of a DMC section and description of their function.
**Rationale for change**		To provided an additional safety measure.
**Section to be changed**		3.3.2 Inclusion criteria
**Description of change**		Clarification to BMI units in inclusion # 5.
**Rationale for change**		Clarification
**Section to be changed**		3.3.3 Exclusion criteria
**Description of change**		Addition of "and/or nitrite" to exclusion # 2.
**Rationale for change**		Clarification
**Section to be changed**		5.1.2.2 Arterial stiffness and sympathetic nervous system (SNS) measurements
**Description of change**		Addition of "as well as a derived aortic AIx" to the first sentence of this section.
**Rationale for change**		Clarification
**Section to be changed**		5.1.2.3 Blood pressure and pulse rate
**Description of change**		Clarifications with respect to the accuracy of data recorded from a manual vs. a digital sphygmomanometer and the selection of the arm for subsequent blood pressure measurements in case a 10 mmHg difference is observed between arms.
**Rationale for change**		Clarification
**Section to be changed**		5.2.2.1 Definitions of adverse events
**Description of change**		Deletion of the words "the investigator and" from the Significant Adverse Event subsection.
**Rationale for change**		Clarification
**Section to be changed**		Table 5.2.3: 1 Summary of laboratory assessments
**Description of change**		The type of hCG assessment under Visits 1, 13 and EOT was clarified and the preferred time point of Visit 1 was corrected from -27 days to -21.
**Rationale for change**		Clarification
**Section to be changed**		Table 5.2.3: 2 Safety and efficacy laboratory parameters – whole blood, serum or plasma

Proprietary confidential information.
© 2012 Boehringer Ingelheim International GmbH or one or more of its affiliated companies. All rights reserved.
This document may not - in full or in part - be passed on, reproduced, published or otherwise used without prior written permission.

**Description of change**		Under the hematology panel, "a relative count" was deleted as it will not be performed. Assessment of hCG added under other assessments.
**Rationale for change**		Clarification
**Section to be changed**		Table 5.2.3: 3 Safety and efficacy laboratory parameters - urine
**Description of change**		Urine hCG deleted from the Quantitative (C) panel and glucose added under this panel. The time point of the 24 hour urine collection was also clarified. Estimated GFR will be derived at all time points when serum creatitine is assessed, as such the words "at screening" were deleted for clarity.
**Rationale for change**		Clarification
**Section to be changed**		7.3.2.2 Other analyses
**Description of change**		Under the "Continuous glucose monitoring analysis" subsection, the words "important" and "secondary" were deleted for clarity.
**Rationale for change**		Clarification

Proprietary confidential information.
© 2012 Boehringer Ingelheim International GmbH or one or more of its affiliated companies. All rights reserved.
This document may not - in full or in part - be passed on, reproduced, published or otherwise used without prior written permission.
